# Physical exercise‐induced circAnks1b upregulation promotes protective endoplasmic reticulum stress and suppresses apoptosis via miR‐130b‐5p/Pak2 signaling in an ischemic stroke model

**DOI:** 10.1111/cns.70055

**Published:** 2024-09-27

**Authors:** Xiaofeng Yang, Yating Mu, Yifeng Feng, Mingyue Li, Haojie Hu, Xiaoya Zhang, Zejie Zuo, Rui Wu, Jinghui Xu, Fang Zheng, Xiaofei He, Xiquan Hu, Liying Zhang

**Affiliations:** ^1^ Department of Rehabilitation Medicine The Third Affiliated Hospital, Sun Yat‐sen University Guangzhou China; ^2^ Department of Psychology New York University New York New York USA

**Keywords:** circAnks1b, ER stress, ischemic stroke, physical exercise

## Abstract

**Aims:**

Physical exercise (PE) can accelerate post‐stroke recovery. This study investigated contributions of circRNAs to PE‐induced improvements in post‐stroke neurological function.

**Methods:**

Rats subjected to transient middle cerebral artery occlusion were left sedentary or provided running‐wheel access for 4 weeks during recovery. CircRNAs from peri‐infarct cortex were identified by high‐throughput sequencing, and interactions with miRNAs by immunoprecipitation, fluorescence in suit hybridization, and dual‐luciferase reporter assays. In vivo circRNA knockdown was achieved using shRNA‐AAVs and in vitro overexpression by plasmid transfection. Transmission electron microscopy, western blotting, and TUNEL assays were conducted to explore circRNA contributions to endoplasmic reticulum (ER) stress and neuronal apoptosis. CircRNA levels were measured in plasma from stroke patients by qRT‐PCR and associations with neurological scores assessed by Pearson's correlation analysis.

**Results:**

PE upregulated circAnks1b, reduced infarct volume, and mitigated neurological dysfunction, while circAnks1b knockdown exacerbated neurological dysfunction and increased infarct size despite PE. CircAnks1b sponged miR‐130b‐5p, thereby disinhibiting Pak2 expression. Conversely, Pak2 downregulation disrupted PE‐mediated protective ER stress, leading to reduced IRE1/XBP1 and heightened apoptosis. Plasma circAnks1b was higher in stroke patients receiving PE than sedentary patients and correlated negatively with neurological scores.

**Conclusions:**

CircAnks1b upregulation may be an effective therapeutic strategy for post‐stroke recovery.

## Graphical abstract

Experimental hypothesis: CircAnks1b is upregulated by physical exercise in neurons of peri‐infarct cortex. CircAnks1b competitively binds to miR‐130b‐5p, thereby mitigating miR‐130b‐5p‐mediated suppression of Pak2 mRNA expression. Ensuing Pak2 expression increases IRE1a/XBP1 signaling pathway activity, which promotes protective ER stress in neurons and reduces apoptosis. High plasma circAnks1b expression also predicts superior neurological recovery in IS patients.

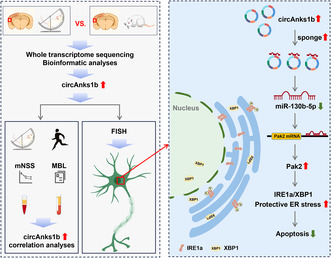

## 1 INTRODUCTION

Stroke is the second most common cause of death worldwide, accounting for 11.6% of all fatalities, and ranks third as a cause of death or disability.^1^ According to the most recent American Heart Association (AHA) conference, about 87% of strokes are ischemic strokes (IS).^2^ Ischemia and reperfusion can induce neuroinflammation, oxidative stress, glutamate excitotoxicity, and various other pathogenic processes^3^ in the peri‐infarct area, leading to neuronal death and associated loss of neurological function. Post‐stroke therapies with some efficacy for accelerating neurological recovery include physical rehabilitation, some select drugs, non‐invasive brain stimulation, and physical exercise (PE). Among these, PE has numerous unique advantages, including low cost and convenience for in‐home care.^4^, ^5^, ^6^ Neurons are highly sensitive to ischemia, and much research on IS has focused on neuropathogenic and neuroprotective mechanisms. Circular (circ)RNAs are relatively stable multifunctional single‐stranded RNA species implicated in the regulation of neuronal growth and differentiation as well as rehabilitation outcome.^7^ The objective of the present study is to explore potential molecular mechanisms contributing to the benefits of PE for IS rehabilitation, including the actions of circRNAs.

Recent studies demonstrated that non‐coding RNAs (ncRNAs), including long non‐coding RNAs (lncRNAs), microRNAs (miRNAs), and circular RNAs (circRNAs), participate in numerous physiological and pathological processes. CircRNAs form from linear RNAs through back‐splicing, connecting the downstream 5′ end to the upstream 3′ end. As these circRNAs are much more stable and more highly conserved than other ncRNAs, they are considered suitable biomarkers for various diagnostic, prognostic, and disease monitoring applications.^8^ In fact, alterations in circRNA expression profiles are associated with the severity and progression of several diseases, including cardiac hypertrophy, chronic obstructive pulmonary disease (COPD), and vascular cognitive impairment (VCI).^9^, ^10^, ^11^ Also, circRNAs appear to regulate neuronal death, microglial polarization, and neuroinflammation,^12^, ^13^, ^14^, ^15^, ^16^ suggesting potential functions in neural recovery after IS. If so, associations would be expected between circRNA expression profiles and the outcome of IS rehabilitation. Furthermore, circRNAs may influence recovery by contributing to neuroprotective processes activated by rehabilitation interventions such as PE. While the majority of previous studies have concentrated on identifying circRNAs for early diagnosis in acute ischemic stroke (AIS) patients, few have investigated contributions to therapeutic recovery. Such studies could identify specific circRNAs as effective therapeutic targets.

CircRNAs have been reported to modulate apoptosis, autophagy, blood–brain barrier (BBB) integrity,^17^, ^18^, ^19^, ^20^, ^21^ angiogenesis, white matter integrity, and neural proliferation, all of which may contribute to PE‐induced recovery after IS.^4^, ^22^, ^23^, ^24^ In addition, circRNAs were recently implicated in endoplasmic reticulum (ER) stress. The ER provides an intracellular compartment specialized for protein synthesis and folding, as well as calcium storage and regulation.^25^ Following IS, the ER accumulates unfolded and improperly folded proteins, which induces ER dysfunction (termed ER stress) and activates the unfolded protein response (UPR).^26^ Sustained or intense ER stress has the potential to trigger cell apoptosis. Conversely, mild ER stress can suppress the production and improper folding of proteins and bolster their degradation, thus preserving cellular equilibrium and fostering cell viability. However, it is unknown if circRNAs also protect against ER stress and if this mechanism contributes to the therapeutic efficacy of PE. Herein, we examined if circRNAs are altered by PE and contribute to the rehabilitative efficacy of PE by reducing ER stress and apoptosis in the transient middle cerebral artery occlusion (tMCAO) model of IS.

## 2 MATERIALS AND METHODS

### 2.1 Animal model and exercise training

A total of 170 male Sprague Dawley rats (220–240 g) were purchased from Guangzhou Qingle Life Science Co., and housed in a specific pathogen‐free environment at 26°C under a 12 h/12 h light/dark cycle with food and water ad libitum. Rats were anesthetized using 1.5% pentobarbital sodium (5 mL/kg body weight, i.p.) and subjected to left tMCAO for 90 min using intraluminal filaments (Beijing Cinontech Co., Ltd., China) by one investigator blinded to subsequent experiments.^27^ The success of tMCAO was confirmed by Bederson score 24 h after the procedure.^28^ In addition, five rats were anesthetized without tMCAO to serve as a sham group for the entire study. Animals with successful tMCAO were randomly divided into PE and sedentary groups. Following our previous study, the PE group rats initiated moderate intensity exercise 48 h after tMCAO using a running wheel (Patent no. CN207544032U).^22^ Moderate intensity was defined as 3 rpm for 20 min twice per day for the first 2 days, gradually increasing to 5 rpm on the third to sixth day and 10 rpm from the seventh day until the end of 4‐week intervention period. Rats in the sedentary group were house under the same conditions but not granted access to a running wheel. All experimental procedures were approved and supervised by the Animal Research Ethics Committee of Lani Scientific Co., Ltd. (Guangzhou, China; approval ID: G2022015).

### 2.2 Human plasma collection

IS patients were recruited from the Department of Rehabilitation at the Third Affiliated Hospital of Sun Yat‐Sen University between February 2023 and September 2023, and randomly divided into IS control and IS plus PE groups. Patients in the PE group walked for 30 min per day for 4 weeks under the guidance of experienced physical therapists as moderate intensity exercise (64%–76% of individual maximum heart rate (HRmax) as calculated by the formula HR_max_ = 207−[0.7 × age]).^29^ Changes in neurological function were assessed using the modified Barthel index (MBI). Study sample size was determined by a preliminary experiment with 3 IS plus PE and 7 IS control group patients. Based on the estimated difference in MBI, 9 cases were needed per group to achieved *α* = 0.05 and *β* = 0.1 by two‐sided independent samples *t*‐test. For the experiment, 10 IS control group patients and 9 IS plus PE group patients were included. This study was approved by the Ethics Committee of the Third Affiliated Hospital of Sun Yat‐Sen University (approval ID: II2023‐034‐01) and conducted in strict accordance with the Declaration of Helsinki. Informed consent was obtained from all participants or their legal representatives.

### 2.3 Quantification of infarct volume

Infarct volume was measured 28 days after tMCAO (immediately following the 4‐week intervention period) by MRI (Bruker Biospin, PharmaScan 70/16, US) or 2,3,5‐triphenyl‐tetrazolium chloride (TTC) staining. For MRI, rats were anesthetized with 2% isoflurane and fixed in a prone position. Following a scout view, T2‐weighted images were acquired using a fast spin‐echo sequence with the following settings: 0.8 mm slice thickness, 33 ms echo time, and 2500 ms repetition time. For TTC staining, the brain was rapidly removed and sliced into 2 mm‐thick sections. Slices were then stained with a 2% TTC solution at 37°C for 30 min. Infarct volume was measured from the same five MRI and TTC slices using ImageJ software and the equation (VC−VI)/VC × 100%, where VI and VC are ipsilateral and contralateral hemisphere volumes, respectively.

### 2.4 Behavioral tests of the neurological function

Neurological function in rats was assessed using the modified neurological severity score (mNSS), ladder rung walking task, and cylinder test.^30^, ^31^ The mNSS evaluation consists of five parts: motor test, placing rat on the floor, sensory test, beam balance test, and reflexes test. Total score ranges from 0 to 18, with high scores indicating more severe dysfunction. Only rats with moderate injury (score 7–12) 24 h post‐tMCAO were included in subsequent experiments.^30^

The ladder rung walking task, which measures deficits and recovery in sensorimotor functions, was conducted using a horizontal ladder, about 100 cm long and 8 cm wide, suspended 30 cm above a platform, with an irregular rung arrangement.^31^ Briefly, each rat was habituated prior to the first test session by placing it on the same rung. Three test sessions were conducted and videotaped for gait analysis as the animal traversed the ladder. Any slight slips, deep slips, and total rung misses were scored as errors, and the error score (ES) was calculated as the ratio of total errors to total steps.

The cylinder test was implemented inside a clear glass cylinder 20‐cm in diameter and 30‐cm in height. Behavior was recorded by a camera for off‐line analysis of forelimb motion.^32^ The numbers of weight‐bearing touches to the cylinder walls with the left, right, or both forelimbs during rearing were recorded up to 20 in total, and the ratio of right to (left + right) touches was calculated as an index of unilateral impairment.

### 2.5 Whole transcriptome sequencing

The peri‐infarct region was excised with RNase‐free instruments under stereoscopy and preserved in RNALater Stabilization solution (AM720, ThermoFisher) for subsequent RNA‐seq analysis by Guangzhou Epibiotek Co., Ltd. (China). Differentially expressed genes (DEGs) were defined by *p* value <0.05 and log_2_∣fold change (FC)∣ ≥ 1. Volcano and heatmap plots were drawn using *R* software.

### 2.6 Gene enrichment analysis

Functions and pathways of DEGs were retrieved from the Gene Ontology (GO) database and Kyoto Encyclopedia of Genes and Genomes (KEGG). Gene set enrichment analysis (GSEA) was conducted using *R* software. All gene expression lists were uploaded, and GO annotations were segregated into biological process (BP), molecular function (MF), and cellular component (CC) subgroups.

### 2.7 Prediction of circRNA–miRNA and miRNA–mRNA interactions

Potential target miRNAs of individual circRNAs were predicted using miRanda and RNAhybrid running on the Linux system, while miRanda, RNAhybrid, and Targetscan (https://www.targetscan.org/vert_80/) were utilized to predict the potential interactions between miRNAs and mRNA targets. Utilizing a comparative approach, the predicted miRNA and mRNA candidates were intersected with their corresponding profiling datasets to delineate a refined set of putative targets. This intersection was graphically represented through Venn diagrams, enabling the prioritization of the most plausible miRNAs and mRNAs for subsequent investigation.

### 2.8 RNA preparation and quantitative real‐time PCR (qRT‐PCR)

Peripheral blood was drawn from anesthetized rats by cardiac puncture into anticoagulant tubes on ice. Samples were then gently inverted and centrifuged at 1500 *g* for 10 min at 4°C to isolate serum. Total RNA (including miRNA) was extracted from rat serum samples as well as from peri‐infarct cerebral cortex lysate and patient plasma using TRIzol reagent (R1200, Solarbio) according to the manufacturer's protocol. Isolated mRNAs were reversely transcribed to cDNAs using the HiScript II 1st Strand cDNA Synthesis Kit (Vazyme) and expression levels quantified by qRT‐PCR with primers designed using Primer‐BLAST online (https://www.ncbi.nlm.nih.gov/tools/primer‐blast). All qRT‐PCR reactions were performed using ChamQ SYBR qPCR Master Mix (Vazyme) on a Roche Light Cycler 96 machine with GAPDH mRNA and U6 miRNA amplified as the endogenous controls. Relative gene expression levels were determined by the 2^−ΔΔ*Ct*^ method. All primer sequences are listed in Table S1.

### 2.9 Sanger sequencing

Rat brain tissue was homogenized and genomic (g)DNA extracted using the FastPure Cell/Tissue DNA Isolation Mini Kit (DC102, Vazyme). Total RNA was extracted and reverse transcribed into cDNA as described in Section 2.8. Target DNA fragments were amplified by PCR using gene‐specific primers (Table S1) and the 2xPhanta Flash Master Mix (P520, Vazyme), purified, size‐fractionated by agarose gel electrophoresis, and sequenced using the Sanger method by IGEbio Co., Ltd., China.

### 2.10 RNA immunoprecipitation assays

RNA immunoprecipitation (RIP) assays were conducted using the BersinBioTM RIP Kit (Bes5101) according to the manufacturer's protocol. In brief, tissue lysates were subdivided into separate input, IP, and IgG samples. The IP sample was incubated with anti‐Ago2 (1:500, A19709, ABclonal) and the IgG sample with rabbit anti‐IgG antibody (Bes5101, 1 μg/μL) at 4°C for 16 h. Subsequently, RNA–protein complexes were captured by incubating these lysates with protein A/G magnetic beads at 4°C for 1 h. After elution, protein‐bound RNAs were extracted with TRIzol reagent (R1200, Solarbio) and quantified by qRT‐PCR as described in Section 2.8.

### 2.11 Stereotactic injections into cortex and lateral ventricles

Vector injections were conducted 14 days before tMCAO to allow for molecular expression based on preliminary results. Rats were anesthetized with 1.5% pentobarbital (5 μL/g, i.p.) and then secured in a stereotaxic apparatus (RWD, Shenzhen, China). The adeno‐associated virus (AAV) sh‐circAnks1b, AAV9 sh‐Pak2, or a corresponding empty vector (all from Vigene Biosciences, Guangzhou, China) was injected into cortex according to a previous study.^33^ The AAV vector or matched empty control vector was injected at AP 0.5, LM 3.5, and Depth − 4 mm, while the AAV9 vector or empty control was injected at AP‐1.9, LM 4, and Depth − 4.5 mm, both at 0.1 μL/min for 10 min. The miR‐130b‐5p agomir (5 μL, RiboBio, Guangzhou, China) and its corresponding miR‐NC were microinjected into the left lateral ventricle (AP 0.9, LM 1.4, Depth − 4 mm), while miR‐130b‐5p antagomir (5 μL, RiboBio) and its corresponding miR‐NC were microinjected into the sh‐circAnks1b site during AAV injection. Needles were removed slowly over 5 min after completion of each injection to ensure adequate diffusion.

### 2.12 Primary cortical neuron culture and the oxygen–glucose deprivation/reoxygenation (OGD/R) model

Cerebral cortices were isolated from anesthetized embryonic rats (aged 16–18 days) following a previously described protocol.^34^ After removal of the meninges and vasculature, tissues were enzymatically dissociated using 0.25% Trypsin–EDTA (25,200,056, Gibco) at 37°C for 20 min, followed by careful trituration. The resulting cell suspensions were seeded onto poly‐l‐lysine‐coated coverslips (24 × 24 mm) and maintained in Neurobasal medium (10888022, Gibco) supplemented with 2% B27 (A3582801, Gibco), 0.5 mM L‐glutamine, and 50 U/mL penicillin/streptomycin under a humidified 5% CO_2_ / 95% O_2_ atmosphere at 37°C. The culture medium was replaced every 48 h. Between the 7th and 9th day in culture, the neurons were washed thrice with phosphate‐buffered saline (PBS) and then transferred into an airtight anaerobic chamber (C‐31, MGC) maintained at 37°C and equipped with an anaerobic culture gas generator (C1, MGC) and anaerobic condition indicator (C‐22, MGC). Cells were treated for 2 h from the time the oxygen content dropped below 0.1%. Subsequently, the culture medium was replaced with fresh normal medium and the cells were returned to the original incubator (37°C with 5% CO_2_/ 95% O_2_) for 12 h. A control group of cells was left untreated.

### 2.13 Cell transfection

Overexpression vectors encoding circAnks1b and Pak2 were constructed by YeShanBio (Guangzhou, China), while miR‐130b‐5p mimic and inhibitor were constructed by RiboBio (Guangzhou, China). Overexpression vectors were transfected into cells using Lipofectamine 2000 (11668019, Invitrogen) in an Opti‐MEM medium (31985070, Invitrogen) according to the manufacturer's instructions, while the miRNA mimics and inhibitor (50 nM) were transfected using Lipofectamine™ RNAiMAX (13778075, Invitrogen) in Opti‐MEM medium according to the manufacturer's instructions.

### 2.14 Dual‐luciferase reporter assay

The putative binding sequences and mutant sequences of rat circAnks1b and Pak2 3′UTRs for miR‐130b‐5p were amplified separately by PCR and inserted into the XhoI/NotI‐digested dual reporter vector pSI‐Check2 (C8021‐1, Promega). Human embryonic kidney 293 T (HEK293T) cells were seeded in 96‐well culture plates for 24 h, then transfected as indicated with wild‐type vector (WT) or mutant vector (MUT) encoding circAnks1b or Pak2 together with the corresponding miR‐mimic or non‐target control (miR‐NC) as indicated (all custom‐designed by IGEbio Co., Guangzhou, China) for 48 h using Lipofectamine 2000 according to the manufacturer's instructions. Luciferase activity driven by the target gene UTR was determined using a Dual Luciferase Assay System (RG027, Beyotime). The activity of firefly luciferase was normalized to that of Renilla luciferase activity for each specimen. All experiments were conducted 3 times with independently transfected cultures.

### 2.15 Western blotting

The penumbra region of cortex was collected 1 month after tMCAO for Western blotting (WB) as described in our previous research.^23^ The primary antibodies used were rabbit anti‐Pak2 (1:1000, A4553, ABclonal), rabbit anti‐ACTB (1:5000, AC038, ABclonal), rabbit anti‐cleaved‐caspase‐3 (1:1000, AF7022, Affinity), rabbit anti‐Bax (1:5000, ab32503, Abcam), and rabbit anti‐Bcl2 (1:1000, AF6139, Affinity). Following overnight incubation with the primary antibodies at 4°C, membranes were incubated with secondary antibodies (1:5000, AS014, ABclonal) at ambient temperature for 1 h. Protein bands were visualized using an ECL kit (KF8003, Affinity) and enhanced chemiluminescence detection system. Protein band density was quantified using ImageJ.

### 2.16 Immunofluorescence staining and fluorescence in situ hybridization (FISH)

The specific steps for Immunofluorescence (IF) are detailed in our previous research.^4^ Briefly, tissues were incubated overnight with one or more of the following primary antibodies: mouse anti‐NeuN (1:200, Millipore, MAB 377), mouse anti‐Iba‐1 (1:400, Servicebio, GB12105), mouse anti‐GFAP (1:400, CST, 3670S), rabbit anti‐Pak2 (1:200, A4553, Abclonal), mouse anti‐Calnexin (1:200, Proteintech, 66,903‐1‐Ig), and mouse anti‐Tomm20 (1:200, Abcam, ab56783). The following day, sections were incubated with secondary antibodies and then mounted in a solution containing 4′,6‐diamidino‐2‐phenylindole (DAPI). For FISH, Cy3‐labeled circAnks1b and FAM‐labeled miR‐130b‐5p probe (YeShanBio, Guangzhou, China) were diluted 1:100 and applied following the manufacturer's protocol. After FISH, brain slices were treated as described for IF. Images were captured using a fluorescence microscope (Leica TSC SP8, Germany) or stimulated emission depletion microscope (STED, STEDYCON‐181902).

### 2.17 TUNEL assay

TUNEL staining was conducted using an apoptosis detection kit (MA0224, Meilunbio) according to the manufacturer's guidelines. Briefly, slices were treated with proteinase K (20 μg/mL) for 10 min, then incubated with TUNEL detection solution in the dark for 60 min at 37°C. Neurons were stained by IF as described in Section 2.16 using the marker rabbit anti‐NeuN (1:300, D3S3I, CST) and double‐positive cells counted.

### 2.18 Transmission electron microscopy

Transmission electron microscopy (TEM) methods were described in our previous study.^4^ Briefly, brain samples were cut into ultrathin slices using a microtome (Leica UC7), counterstained, and observed under a TEM (Tecnai G2 Spirit).

### 2.19 Statistical analyses

All results were analyzed statistically using GraphPad Prism version 8.0.1. Datasets were first tested for normality using the Shapiro–Wilk test. Based on these results (where *p* > 0.05 indicates a normal distribution), all results are expressed as mean ± standard error of the mean (SEM). Behavioral results obtained at multiple time points were compared by two‐way repeated measures analysis of variance (RM‐ANOVA) with post hoc Holm‐Sidak tests for multiple comparisons. Two independent groups were compared by two‐tailed Student's *t*‐test, and more than two groups by one‐way ANOVA followed by Bonferroni's or Tukey's post‐hoc tests for multiple comparisons. Correlations were assessed using a linear regression model (Pearson's correlation *t* test). A *p* value of < 0.05 was considered statistically significant for all tests.

## 3 RESULTS

### 3.1 Moderate PE reduces infarct volume and concomitantly elevates circAnks1b in the peri‐infarct cortex, while circAnks1b knockdown reduces the protective effects of PE

**FIGURE 1:**
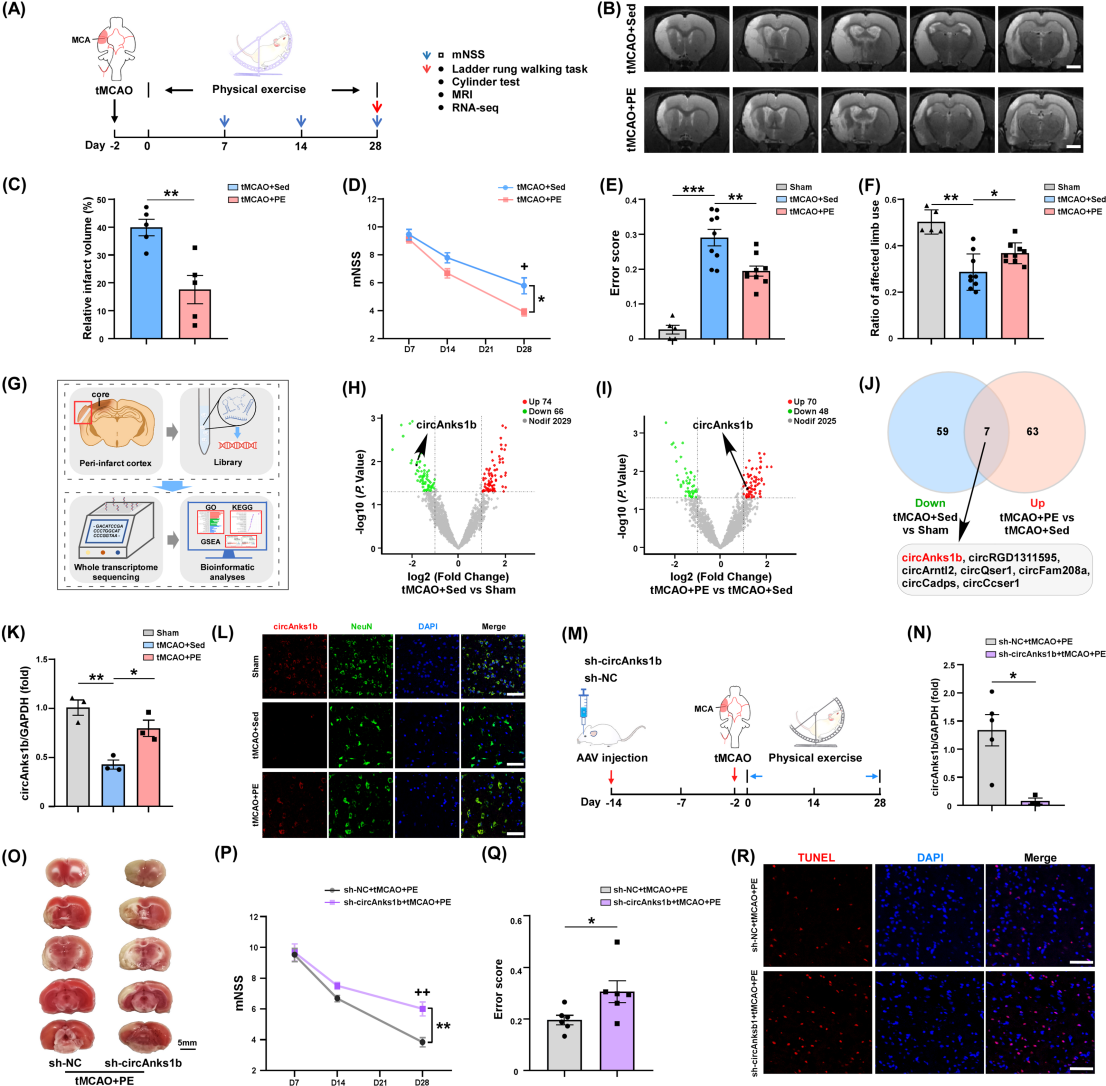
Moderate PE increases circAnks1b expression, reduces infarct volume, and improves neurological recovery among IS model rats, while circAnks1b knockdown reverses these protective effects. (A) Schematic diagrams of experimental time points. (B) T2‐weighted MRI of infarct volume 28 days after transient middle cerebral artery occlusion (tMCAO) and after 28 days of PE or sedentary (Sed) housing (control). (C) Relative infarct volume analysis (*p* = 0.0053). (D–F) Neurological function measured by the modified neurologic severity score or mNSS (D, *p* = 0.0349 at D28), the ladder rung walking task (E, *p* = 0.0034), and cylinder test of forepaw function (F, *p* = 0.0297). (G) Schematic showing whole transcriptome sequencing and bioinformatic analyses. (H) Volcano plot of circRNAs expressed by tMCAO+Sed and Sham groups. (I) Volcano plot of circRNAs expressed by tMCAO+PE and tMCAO+Sed groups. (J) Venn diagram showing overlapping circRNAs. (K) qRT‐PCR analysis of circAnks1b in peri‐infarct cortex of tMCAO+PE, tMCAO+Sed, and sham groups (*p* = 0.0028, 0.0243). (L) FISH assay showing the co‐expression of circAnks1b and a neuronal marker (NeuN) in peri‐infarct cortex. (M) Schematic showing AAV‐injection and experimental time points. (N) qRT‐PCR analysis of circAnks1b in peri‐infarct cortex (*p* = 0.0152). (O) TTC staining of brain tissues after AAV treatment. (P) Difference in mNSS between sh‐circAnks1b and sh‐NC groups (*p* = 0.0097 at D28). (Q) Difference in ladder rung walking task error ratio between sh‐circAnks1b and sh‐NC groups (*p* = 0.0396). (R) TUNEL assay results for sh‐circAnks1b and sh‐NC groups. All results expressed as mean ± SEM. *p < 0.05, **p < 0.01, and ***p < 0.001. +*p* < 0.05, ++*p *< 0.01, tMCAO+PE versus tMCAO+Sed group at different time point. Scale bar is 2 mm (B) or 10 μm (K).

To investigate the potential benefits of PE for accelerating recovery following IS and the underlying molecular mechanisms, we established the rat tMCAO model and conducted a series of neuroimaging and molecular analyses. Acquisition of T2‐weighted magnetic resonance images 4 weeks post‐tMCAO revealed that PE significantly reduced total infarct volume compared to the sedentary tMCAO+Sed group (Figure 1B,C, *p*= 0.0053). Moreover, mNSS score and ladder rung walking task ES ratio were significantly lower in the tMCAO+PE group than the tMCAO+Sed group (Figure 1D, *p* = 0.0349 at D28; Figure 1E, *p* = 0.0034), while affected limb use was higher in the tMCAO+PE group (Figure 1F, *p* = 0.0297). Thus, PE reduced infarct volume and improved neurological function at 28 days after model IS.

To examined the potential contributions of circRNAs to these improvements in IS recovery, we first conducted bioinformatics analyses of circRNAs based on high‐throughput sequencing. The volcano plot and heatmap in Figure 1G,I illustrate that 74 circRNAs were upregulated and 66 circRNAs were downregulated in peri‐infarct cortex of tMCAO+Sed group rats, while 70 circRNAs were upregulated and 48 were downregulated after PE (Figure 1G–I, Figure S1A,B). Seven circRNAs were identified that were upregulated after PE but downregulated after IS alone (Figure 1J): circAnks1b, circRGD1311595, circArntl2, circQser1, circFam208a, circCadps, and circCcser1. CircAnks1b (full name *circAnks1b* (*3*,*4*,*5*,*6*,*7*,*8*) according to the new guide for circRNA naming^35^) has been reported to modulate multiple brain functions. The change indicated by RNA‐seq was confirmed by qRT‐PCR (Figure 1K, tMCAO+Sed vs. sham, *p* = 0.0028; tMCAO+PE vs. tMCAO+Sed, *p* = 0.0243) and the putative back‐spliced junction (BSJ) fragment verified by PCR amplification with divergent primers and confirmed by Sanger sequencing (Figure S1C). Nucleic acid electrophoresis further verified its circular structure (Figure S1C). We found that circAnks1b was expressed mainly in neurons rather than astrocytes or microglia, and upregulated in neurons of tMCAO+PE rats as evidenced by FISH (Figure S1D; Figure 1L).

To test whether circAnks1b contributes to PE‐enhanced neuronal survival in peri‐infarct cortex, we microinjected sh‐NC or sh‐circAnks1b AAV into the left (ipsilateral) cortex of rats 2 weeks before tMCAO surgery. Quantitative RT‐PCR analysis revealed a marked decrease in circAnks1b expression within the peri‐infarct cortex following sh‐circAnks1b AAV injection but not after sh‐NC injection (Figure 1N, *p* = 0.0152). Moreover, the beneficial effects of PE on post‐tMCAO recovery were largely abolished by sh‐circAnks1b, as infarct volume was significantly larger than in the sh‐NC + tMCAO+PE group (Figure 1O; Figure S1E, *p* = 0.0203) and all neurological function metrics were poorer than in the sh‐NC + tMCAO+PE group at D28 (Figure 1P, *p* = 0.0097 for mNSS at D28; Figure 1Q, *p*= 0.0396 for the ladder rung walking test; Figure S1F, *p* = 0.0026 for the cylinder test). Furthermore, TUNEL assays showed that sh‐circAnks1b injection prior to tMCAO and PE increased the number of apoptotic cells in peri‐infarct cortex compared to sh‐NC injection (Figure 1R). Collectively, these findings strongly suggest that the exercise‐induced upregulation of circAnks1b contributes to PE‐enhanced neuronal survival and functional recovery after IS.

### 3.2 CircAnks1b serves as a sponge for miR‐130b‐5p, reducing its levels post‐exercise in the peri‐infarct cortex

**FIGURE 2:**
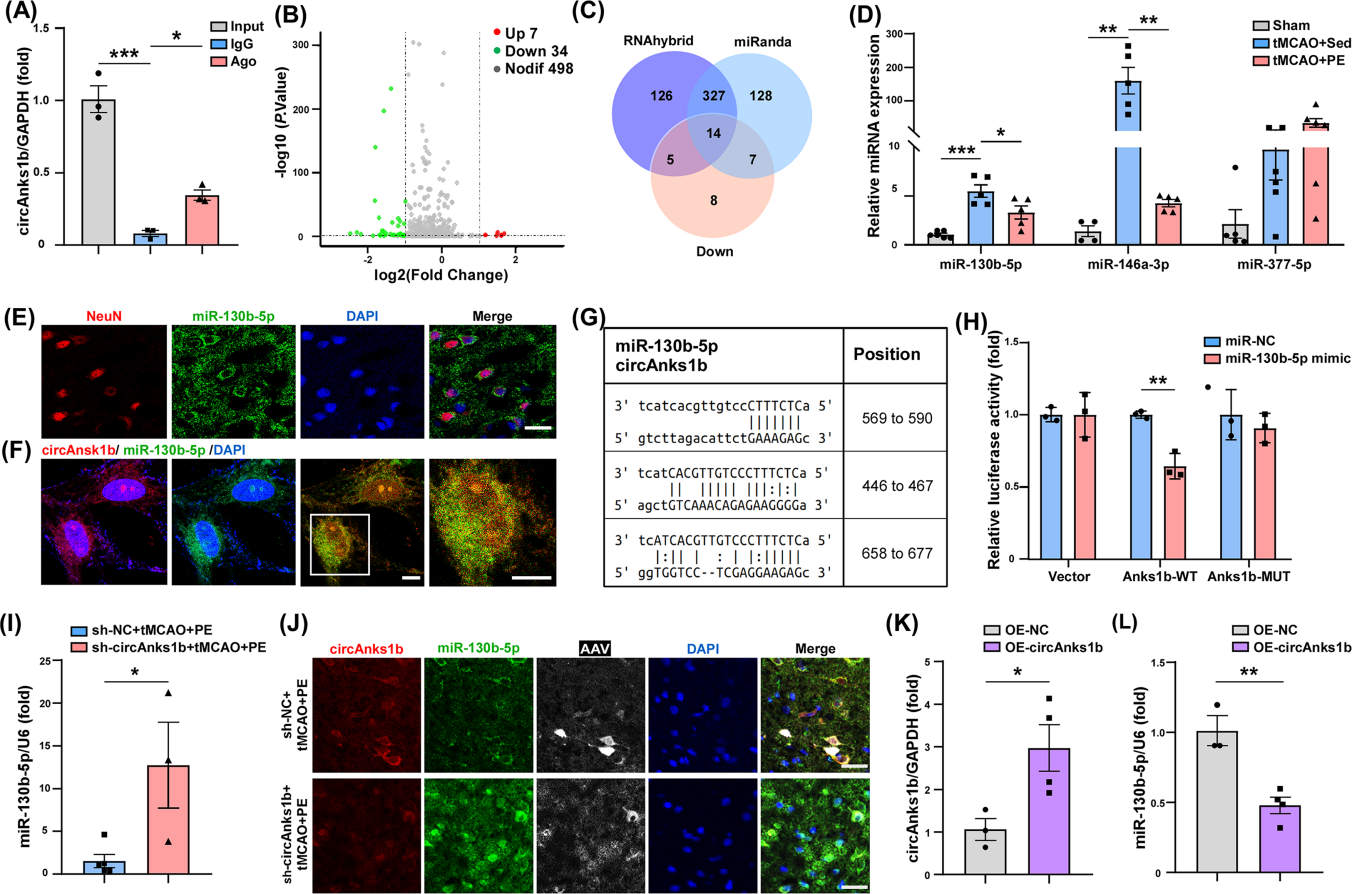
CircAnks1b sponges miR‐130b‐5p in the peri‐infarct cortex. (A) Analysis of CircAnks1b and Ago2 interaction via RIP assay with anti‐Ago2 antibody (*p* < 0.0001, *p* = 0.0417). (B) Volcano plot of miRNAs expressed by tMCAO+PE and tMCAO+Sed groups. (C) Venn diagram showing overlapping miRNAs. (D) qRT‐PCR analysis of miR‐130b‐5p, miR‐146a‐3p, and miR‐377‐5p in peri‐infarct cortex. (E) Fluorescence in situ hybridization (FISH) revealing miR‐130b‐5p expression in peri‐infarct cortex neurons. (F) FISH demonstrating colocalization of circAnks1b and miR‐130b‐5p in primary neurons. (G) Potential interaction sites between circAnks1b and miR‐130b‐5p. (H) Luciferase reporter assay of 293 T cells co‐transfected with Anks1b‐WT or Anks1b‐MUT plus miR‐130b‐5p mimic or miR‐NC (*p* = 0.0062). (I) qRT‐PCR analysis of miR‐130b‐5p in peri‐infarct cortex (*p* = 0.0262). (J) FISH assay for circAnks1b and miR‐130b‐5p in peri‐infarct cortex. (K) qRT‐PCR analysis of circAnks1b in primary rat neurons (*p* = 0.0382). (L) qRT‐PCR analysis of miR‐130b‐5p in rat primary neurons (*p* = 0.0054). All results expressed as mean ± SEM. *p < 0.05, **p < 0.01, and ***p < 0.001. Scale bar is representative of 5 μm (E);10 μm (F, J). WT, wide type; MUT, mutant.

Fluorescence in situ hybridization revealed circAnks1b expression mainly in the neuronal cytoplasm (Figure 1L), where circRNAs primarily regulate mRNAs by targeted sponging of miRNAs.^36^ Moreover, Ago2 RIP assays suggested that circAnks1b acts as a competing endogenous (ce)RNA (Figure 2A). Volcano and heat maps generated by miRNA‐seq of the peri‐infarct zone revealed 7 miRNAs upregulated and 34 downregulated in tMCAO+PE compared to tMCAO+Sed group rats (Figure 2B, Figure S2A) while miRanda and RNAhybrid programs predicted that of the 14 downregulated miRNAs screened (Figure 2C, Figure S2B), three (miR‐130b‐5p, miR‐377‐5p, and miR‐146a‐3p) have more binding sites for circAnks1b. Also, qRT‐PCR showed that expression changes in miR‐130b‐5p and miR‐146a‐3p differed significantly between tMCAO+PE and tMCAO+Sed groups (Figure 2D, *p*= 0.0276 for miR‐130b‐5p, *p* = 0.0020 for miR‐146a‐3p). A previous study also reported circAnks1b/miR‐146a binding in the hippocampus of chronic unpredictable mild stress (CUMS) model rats.^37^ In the present study, FISH revealed miR‐130b‐5p expression predominantly in the neuronal cytoplasm (Figure 2E, Figure S2C) as well as colocalization with circAnks1b (Figure 2F). Furthermore, transfection of a miR‐130b‐5p mimic markedly diminished the luciferase activity of the wild‐type (WT) circAnks1b reporter but not a mutant (MUT) reporter in 293 T cells (Figure 2G,H, *p* = 0.0062). These qRT‐PCR and FISH results strongly suggest reciprocal regulation of circAnks1b and miR‐130b‐5p, with circAnks1b upregulation suppressing free miR‐130b‐5p accumulation (Figure 2I, *p*= 0.0262; Figure 2J). To validate this molecular interaction at the cellular level, we established a cellular model of stroke. Exposure of primary rat neurons to oxygen–glucose deprivation with reperfusion (OGD/R), an in vitro model of ischemia, markedly upregulated circAnks1b and downregulated miR‐130b‐5p (Figure 2K, *p* = 0.0382; Figure 2L, *p* = 0.0054; Figure S2E), providing further evidence that circAnks1b may act as a molecular sponge for miR‐130b‐5p within neuronal cells.

To explore the potential involvement of miR‐130b‐5p in the pathophysiology of IS, we examined the effect of upregulation by intracerebroventricular injection of miR‐130b‐5p agomir 2 weeks before tMCAO surgery. Upregulation as confirmed by qRT‐PCR (Figure S3A, *p* = 0.0016) led to more pronounced neurological impairments compared to miR‐NC injection after 4 weeks of PE in tMCAO model rats (Figure S3B–D, *p* = 0.0156 for the mNSS at D28, *p* = 0.0133 for the ladder walking test, *p* = 0.0129 for the cylinder test). Furthermore, TTC staining revealed larger infarct volumes in the miR‐130b‐5p agomir group of tMCAO+PE rats compared to the miR‐NC group at 4 weeks post‐IS (Figure S3E, *p* = 0.0432). These findings suggest that suppression of the miR‐130b‐5p expression by circAnks1b upregulation contributes to the benefits of PE on post‐IS recovery. We also wondered whether blocking the up‐regulated level of miR‐130b‐5p will ameliorate the damage brought by decreased circAnks1b or not. There was no significant difference in circAnks1b expression between the sh‐circAnk1b and sh‐circAnks1b + antagomi‐miR‐130b‐5p groups (Figure S3F), while there was a notable reduction in miR‐130b‐5p levels in the group treated with sh‐circAnks1b in combination with anta‐miR‐130b‐5p (Figure S3G, *p* = 0.0004). Behavioral tests also confirmed that blocking miR‐130b‐5p elevation under circAnk1b suppression promoted the recovery of neurological function in tMCAO rats (Figure S3H–J, *p* = 0.0141 for the mNSS at D28, *p* = 0.0471 for the ladder rung walking test, *p* = 0.0293 for the cylinder test). TTC staining also revealed that sh‐circAnks1b + anta‐miR‐130b‐5p reduced infarct volume (Figure S3K, *p* = 0.0393). Collectively, these findings indicate that circAnks1b functions through miR‐130b‐5p.

### 3.3 Pak2, a functional target of miR‐130b‐5p, contributes to PE‐associated ER stress

**FIGURE 3:**
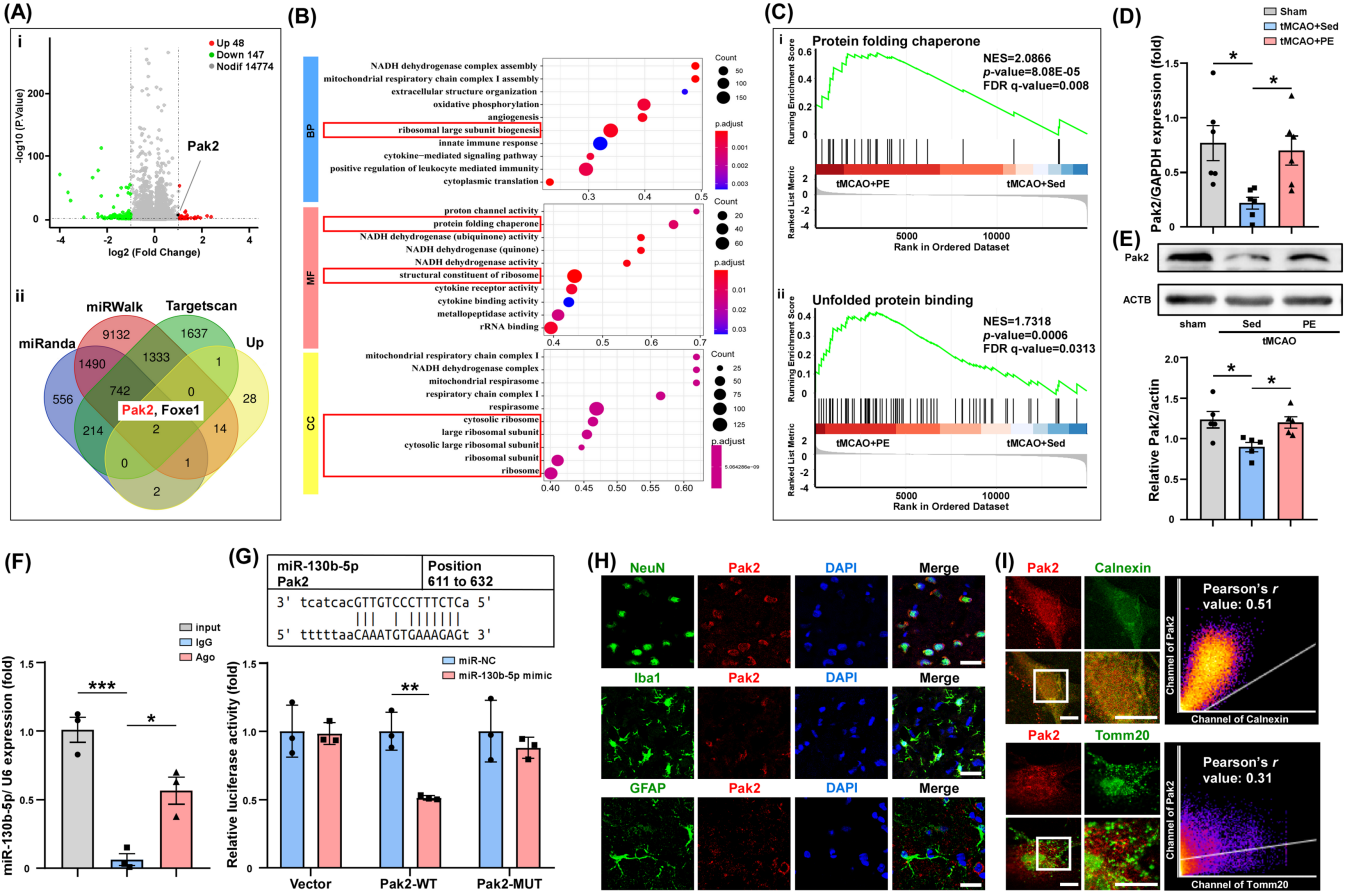
Pak2 is a functional target of miR‐130b‐5p and associated with protective ER activities during exercise. (Ai) Volcano plot of mRNAs expressed by tMCAO+PE and tMCAO+Sed groups. (Aii) Venn diagram depicting common mRNAs. (B) Gene Ontology results from GSEA analyses. (C) Two ER‐relevant KEGG results from GSEA analyses. (D) qRT‐PCR analysis of Pak2 in peri‐infarct cortex. (E) Western blot assessment of Pak2 protein expression in peri‐infarct cortex. (F) RIP experiment with anti‐Ago2 antibody to investigate miR‐130b‐5p and Ago2 interaction (*p* = 0.0417, IgG vs. Ago). (G) Luciferase reporter assay of 293 T cells with Pak2‐WT or Pak2‐MUT co‐transfected with miR‐130b‐5p mimic or miR‐NC (*p* = 0.0035). (H) Immunofluorescence showing the colocalization of Pak2 with a neuronal marker (NeuN) but not an astrocyte marker (GFAP) or microglial marker (Iba1) in peri‐infarct cortex. (I) Super‐resolution STED showing the colocalization of Pak2 and an ER marker (Calnexin) and a mitochondrial marker (Tomm20). *p < 0.05, **p < 0.01, and ***p < 0.001. Scale bar is representative of 5 μm (K) and 20 μm (L). WT: Wide type; MUT: Mutant.

Next, to delineate how circAnks1b/miR‐130b‐5p interaction modulates IS pathology, we identified mRNAs potentially regulated by the observed reciprocal changes in circAnks1b/miR‐130b‐5p expression. Applying the thresholds of *p* < 0.05 and log_2_ (FC) >1, we identified 195 DEGs at the mRNA level, with 48 upregulated and 147 downregulated (Figure 3Ai and Figure S4A). By combining the upregulated mRNAs and those potentially regulated by miR‐130b‐5p, miRanda, miRWalk, and Targetscan identified two mRNAs: Pak2 (encoding p21 (RAC1) activated kinase 2) and FOXE1 (encoding forkhead box E1) (Figure 3Aii).

GO analysis and whole transcriptome sequencing revealed that most pathways significantly altered by PE were related to ER activity (Figure 3B), and GSEA based on the expression matrix of all genes indicated that ER stress pathways, like “protein folding chaperone” and “UPR” were activated by PE (Figure 3C). Consistent with GO analysis, Pak2, a serine/threonine kinase and a key effector of cytoskeletal recombination and nuclear signaling, has been identified as a novel ER stress response factor with documented cytoprotective efficacy.^38^, ^39^ Moreover, consistent with RNA‐seq results, PE increased the expression of Pak2 at the mRNA and protein levels (Figure 3D, tMCAO+PE vs. tMCAO+Sed group, *p* = 0.0376; Figure 3E, *p* = 0.0493). Furthermore, Ago2‐RIP assay indicated that miR‐130b‐5p was enriched by anti‐Ago2 pull‐down of peri‐infarct lysate compared to anti‐IgG (Figure 3F, *p* = 0.0115) and dual‐luciferase reporter assays showed that miR‐130b‐5p mimic reduced the luciferase activity driven by Pak2 WT 3′UTR but not Pak2 MUT 3′UTR (Figure 3G, *p* = 0.0035; Figure S4B). Also, IF showed that Pak2 was located mainly in rat cortical neurons rather than astrocytes and microglial cells (Figure 3H), and super‐resolution STED microscopy localized Pak2 in closer proximity to the ER membrane (Pearson's *r* = 0.51) than mitochondrial membrane (Pearson's *r* = 0.31) (Figure 3I). Collectively, these findings indicate that Pak2 is directly targeted by miR‐130b‐5p and that Pak2 may contribute to the ER function.

### 3.4 Pak2 contributes to PE‐enhanced recovery via circAnks1b/miR‐130b‐5p pathway upregulation

**FIGURE 4:**
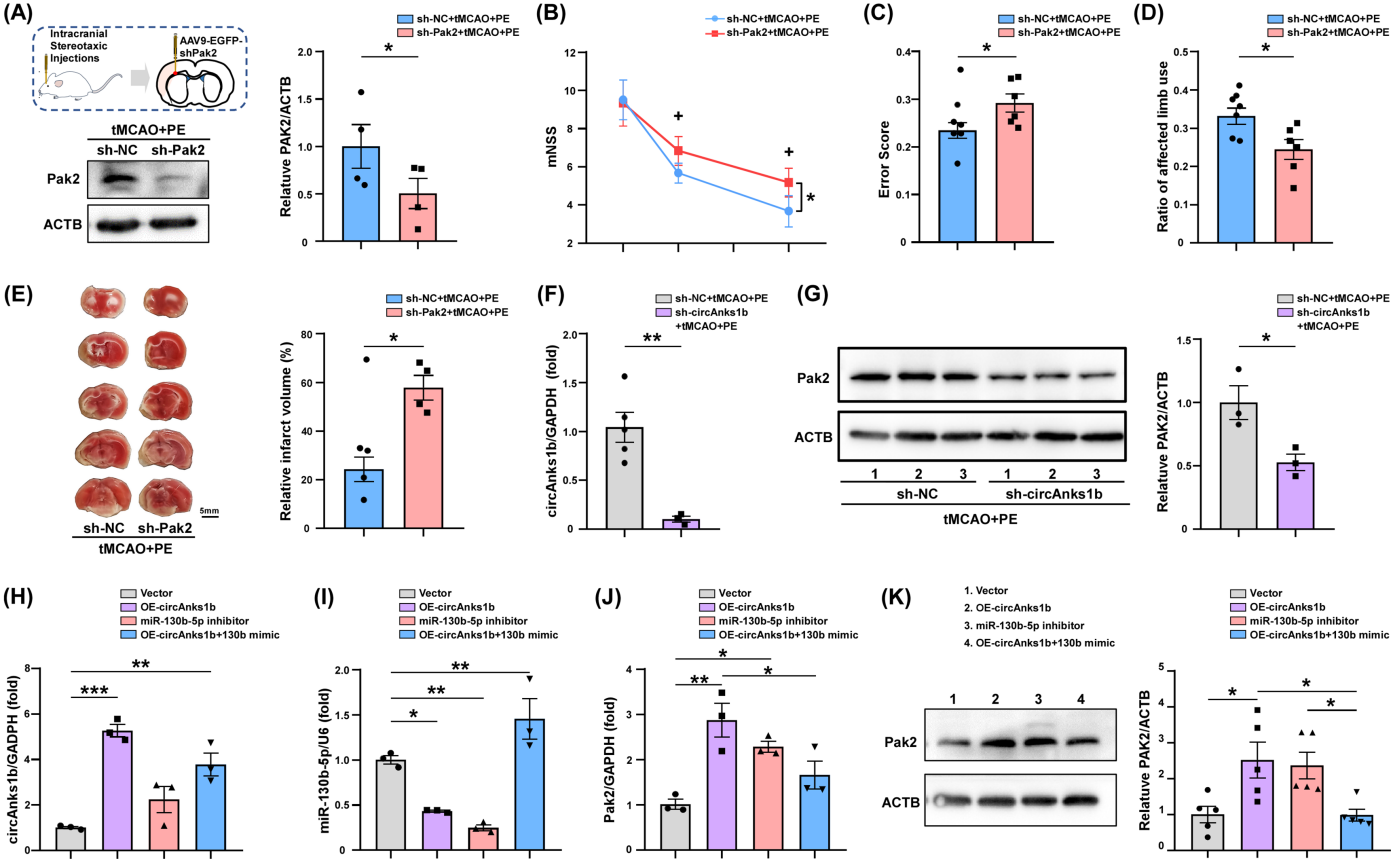
Pak2 is regulated by the circAnks1b/miR‐130b‐5p pathway. (A) Schematic diagrams of intracranial stereotaxic injections and western blotting to confirm AAV efficiency and Pak2 expression changes in peri‐infarct cortex (*p* = 0.0260). (B–D) Neurological function measured by the mNSS (B, *p* = 0.0237), ladder rung walking task (C, *p* = 0.0463), and cylinder test (D, *p* = 0.0251). (E) TTC staining of brain tissues after sh‐NC or sh‐Pak2 treatment, and quantification of brain infarct volume (*p* = 0.0033). (F) qRT‐PCR analysis of Pak2 mRNA expression in peri‐infarct cortex of sh‐NC and sh‐circAnks1b groups (*p* = 0.0037). (G) Western blot detection of Pak2 protein expression in peri‐infarct cortex of sh‐NC and sh‐circAnks1b groups (*p* = 0.0336). (H–J) qRT‐PCR evaluation of circAnks1b, miR‐130b‐5p, Pak2 mRNA expression in primary rat neurons from specified groups. (K) Western blot analysis of Pak2 protein expression in primary rat neurons of the indicated groups. *p < 0.05, **p < 0.01, and ***p < 0.001.

To examine if PE‐induced Pak2 upregulation actually contributes to neurological recovery, we induced AAV9‐mediated Pak2 gene silencing 2 weeks before tMCAO by injection of U6 promoter‐driven sh‐Pak2 (4.21 × 10^13^ Vg/mL) or AAV9‐EGFP (as the control). Western blotting confirmed that Pak2 shRNA successfully inhibited Pak2 expression in cortex (Figure 4A, *p* = 0.0260) and concomitantly abolished the benefits of PE as evidenced by both poorer neurological test performance (Figure 4B–D, *p* = 0.0237 for the mNSS at D28; *p* = 0.0463 for the ladder rung walking test; *p* = 0.0251 for the cylinder test) and larger infarct size (Figure 4E, *p* = 0.0033). Furthermore, the increased Pak2 expression in tMCAO+PE rats was reduced at mRNA and protein levels by circAnks1b knockdown (Figure 4F, *p* = 0.0037; Figure 4G, *p* = 0.0336).

We then verified this relationship between circAnks1b/miR‐130b‐5p and Pak2 in primary neurons. Primary cultured rat neurons were transfected with the indicated vectors 24 h before and harvested 48 h after OGD/R. Upregulation of circAnks1b led to a significant reduction in miR‐130b‐5p expression and an elevation in Pak2 expression, and miR‐130b‐5p inhibitor treatment also upregulated Pak2. Rescue assays confirmed that miR‐130b‐5p suppression is the essential mediator of Pak2 upregulation, as miR‐130b‐5p overexpression attenuated circAnks1b overexpression‐induced Pak2 mRNA upregulation (Figure 4H–J) and protein upregulation (Figure 4K). These data suggest that neuronal Pak2 is negatively regulated by miR‐130b‐5p and that circAnks1b acts as a sponge to downregulate miR‐130b‐5p, leading to disinhibition of Pak2 expression both in peri‐infarct rat cortex and primary rat neurons. Moreover, only Pak2 overexpression independently reduced cell apoptosis following OGD/R.

### 3.5 Pak2 knockdown disrupts exercise‐induced protective ER stress and increases neuronal apoptosis

**FIGURE 5:**
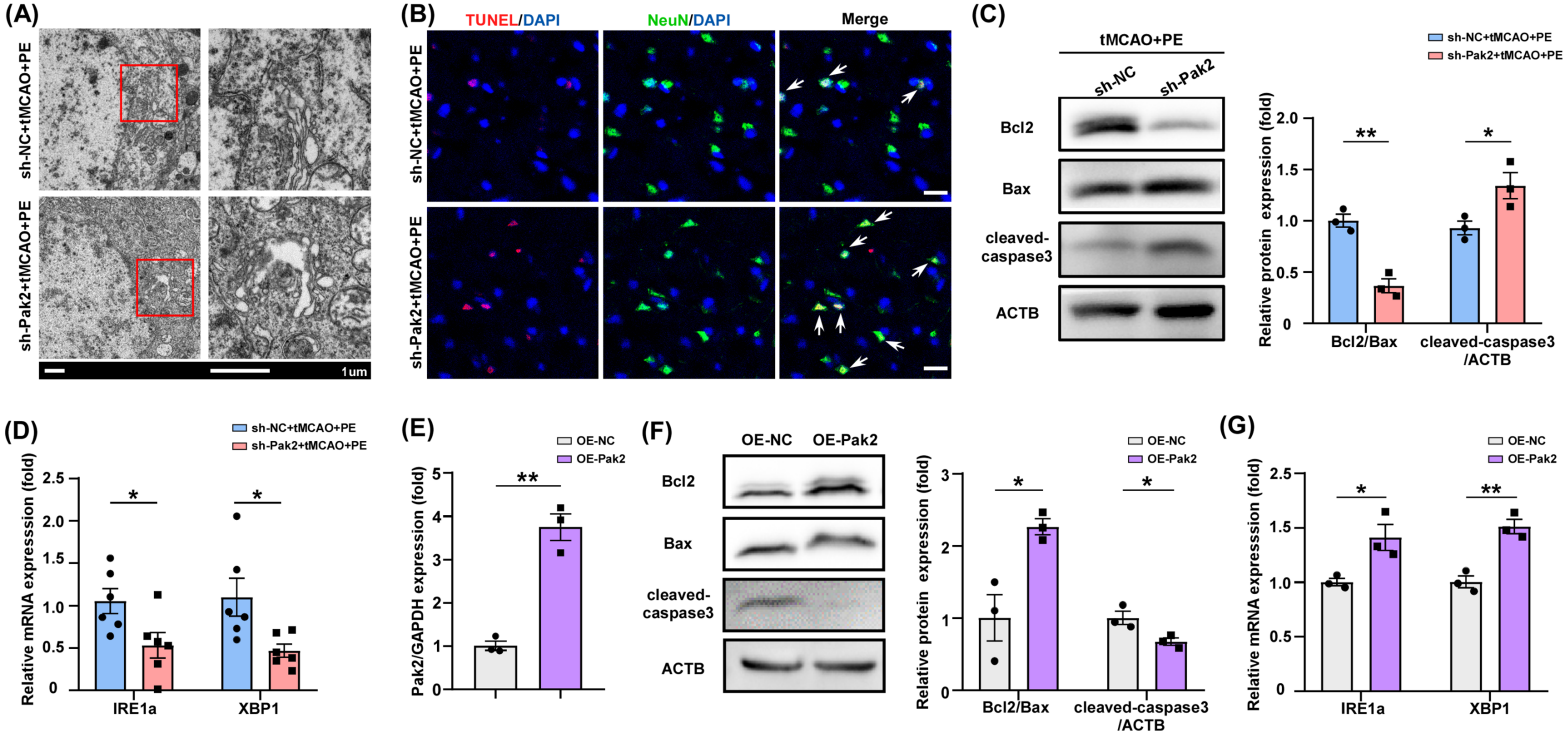
Pak2 suppression disrupts exercise‐induced protective ER stress and promotes neuronal apoptosis. (A) TEM images of ER in peri‐infarct cortex. (B) TUNEL assay showing neuronal apoptosis in sh‐NC and sh‐Pak2 groups. (C) Western blot analysis of Bcl2, Bax, and cleaved‐caspase‐3 expression levels in peri‐infarct cortex of sh‐NC and sh‐Pak2 groups (*p* = 0.0023, 0.0457). (D) qRT‐PCR analysis of Pak2 expression in peri‐infarct cortex of sh‐NC and sh‐Pak2 groups (*p* = 0.0327, 0.0234). (E) qRT‐PCR analysis of Pak2 expression in ischemic neurons transfected with OE‐NC or OE‐Pak2 plasmid (*p* = 0.0011). (F) Western blot analysis of Bcl2, Bax, and cleaved‐caspase‐3 in ischemic neurons (*p* = 0.0203, 0.0354). (G) qRT‐PCR analysis of IRE1a and XBP1 expression in ischemic neurons (*p* = 0.0301, 0.0041). *p < 0.05 and **p < 0.01. Scale bar is 10 nm (A) and 20 μm (B).

A previous study reported that Pak2 preserves ER function through the inositol‐requiring enzyme 1α (IRE1α)/X‐box binding protein 1 (XBP1) signaling pathway.^39^ so we examined if Pak2 upregulation in peri‐infarct cortex by PE also enhances ER function following IS. TEM revealed expanded, balloon‐like ER structures in neurons of tMCAO+PE rats also transfected with sh‐Pak2 compared to rats transfected with sh‐NC (Figure 5A), and TUNEL assay showed that sh‐Pak2 (knockdown) also increased neuronal apoptosis (Figure 5B). The balance between expression of the anti‐apoptotic protein Bcl2 and pro‐apoptotic protein Bax is a decisive factor in determining activation of the intrinsic apoptosis pathway, with a lower Bcl2/Bax ratio increasing proteolytic activation of caspase‐3 (cleaved‐caspase‐3), the primary apoptosis effector. Western blotting confirmed that sh‐Pak2 transfection (Pak2 knockdown) increased cleaved‐caspase‐3 in ischemic brain (Figure 5C, *p* = 0.0023; 0.0457). Consistent with a previous study,^38^ sh‐Pak2 transfection also impaired the expression of the ER stress sensor protein IRE1α and stress‐response transcription factor XBP1 (Figure 5D, *p* = 0.0327 and 0.0234, respectively), which suggests a disruption of the protective ER stress response. These results were further validated at the cellular‐level. Overexpression of Pak2 in OGD/R‐exposed primary neurons (Figure 5E, *p* = 0.0011) increased the Bcl2/Bax ratio and inhibited cleaved‐caspase‐3 expression (Figure 5F, *p* = 0.0203; 0.0354), indicating reduced apoptotic activity, and also enhanced IRE1α and XBP‐1 expression (Figure 5E, *p* = 0.0011; Figure 5G, *p* = 0.0301; 0.0041). These findings imply that the protective effects of Pak2 may be partially attributable to promotion of protective ER stress and a concomitant reduction in neuronal apoptosis.

### 3.6 Exercise‐induced, Pak2‐mediated protective ER stress is regulated by the circAnks1b/miR‐130b‐5p pathway

**FIGURE 6:**
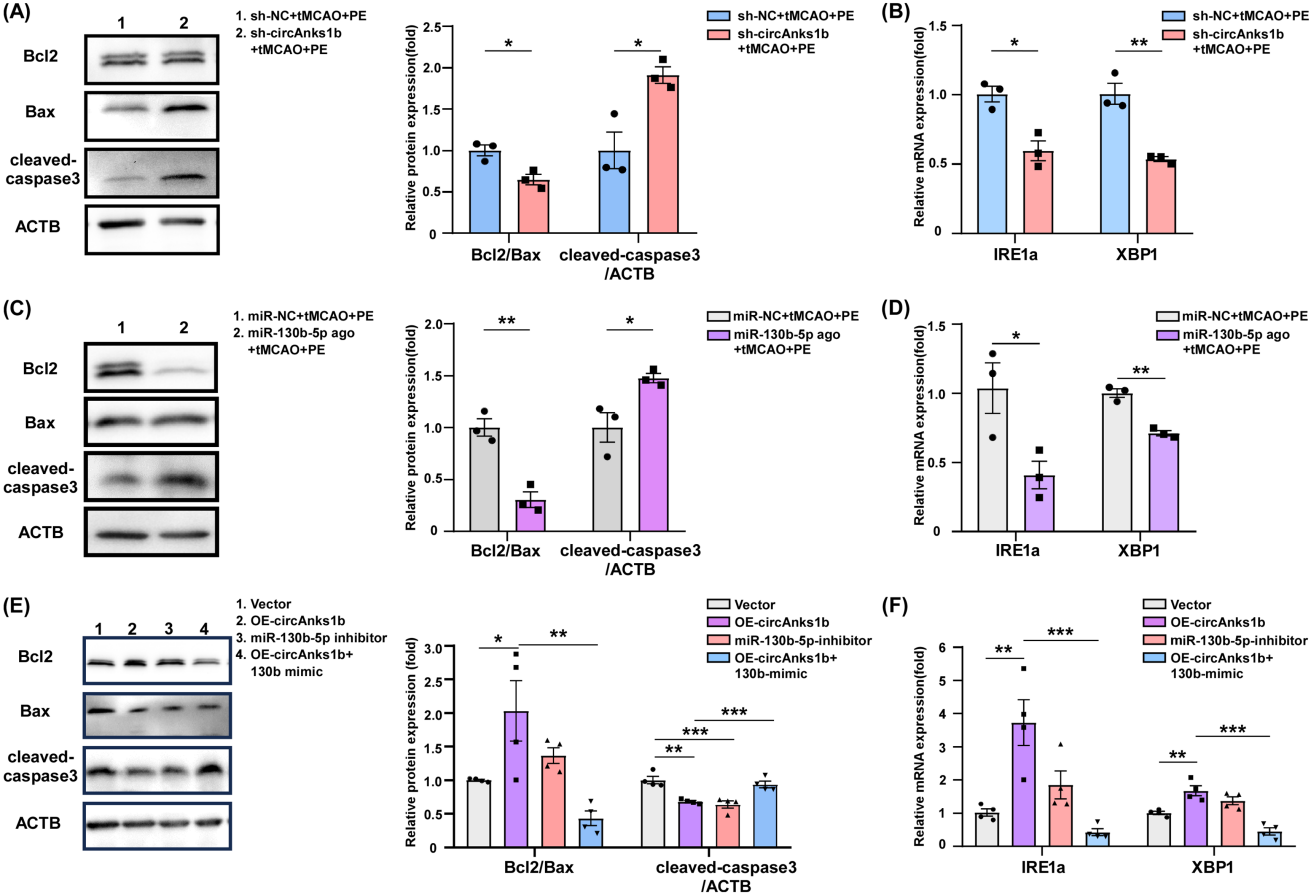
The circAnks1b/miR‐130b‐5p axis regulates Pak2‐mediated protective ER stress induced by exercise. (A) Western blot analysis of Bcl2, Bax, and cleaved‐caspase‐3 expression in peri‐infarct cortex of sh‐NC and sh‐circAnks1b groups (*p* = 0.0182, 0.0200). (B) qRT‐PCR analysis of IRE1a and XBP1 expression in peri‐infarct cortex of sh‐NC and sh‐circAnks1b groups (*p* = 0.0107, 0.0038). (C) Western blot analysis of Bcl2, Bax, and cleaved‐caspase‐3 in peri‐infarct cortex of miR‐NC and miR‐130b‐5p agonist groups (*p* = 0.0034, 0.0337). (D) qRT‐PCR analysis of IRE1a and XBP1 expression in peri‐infarct cortex of miR‐NC and miR‐130b‐5p agonist groups (*p* = 0.0392, 0.0014). (E) Western blot analysis of Bcl2, Bax, and cleaved‐caspase3 expression in ischemic neurons of the indicated groups. (F) qRT‐PCR analysis of IRE1a and XBP1 expression in ischemic neurons of the indicated groups. *p < 0.05, **p < 0.01, and ***p < 0.001.

We then examined if modulating sh‐circAnks1b and miR‐130b‐5p also regulate protective ER stress and apoptosis. Indeed, WB confirmed that both circAnks1b knockdown via sh‐circAnks1b transfection and miR‐130b‐5p upregulation increased apoptosis (Figure 6A, *p* = 0.0182, 0.0200; Figure 6C, *p* = 0.0034, *p* = 0.0337), while IRE1α and XBP1 expression were reduced by both circAnks1b knockdown (sh‐circAnks1b + tMCAO + PE group) (Figure 6B, *p* = 0.0107, 0.0038) and miR‐130b‐5p upregulation (miR‐130b‐5p agonist+tMCAO+PE group) (Figure 6D, *p* = 0.0392, 0.0014). These data suggest that the neuroprotective effects of PE‐induced circAnk1b elevation and concomitant attenuation of miR‐130b‐5p expression are associated with Pak2‐induced promotion of protective ER stress and reduced apoptotic signaling. Furthermore, both circAnks1b overexpression and miR‐130b‐5p knockdown upregulated IRE1a and XBP1 expression levels and reduced apoptosis. Also, the anti‐apoptotic effect of circAnk1b was inhibited by miR‐130b‐5p mimic (Figure 6E,F). Thus, Pak2‐mediated protective ER stress is regulated by the circAnks1b/miR‐130b‐5p axis.

### 3.7 Exercise‐induced plasma circAnks1b elevation is predictive of superior neurological recovery after IS

**FIGURE 7:**
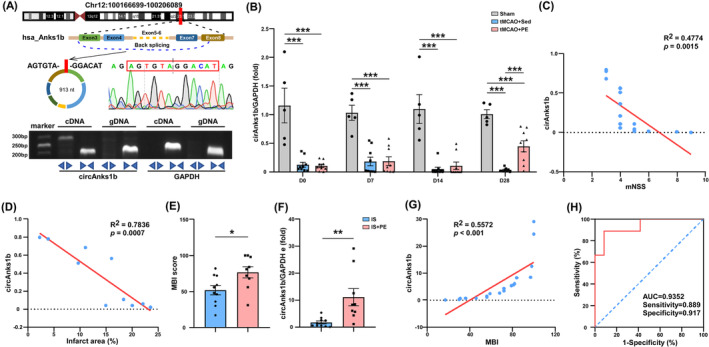
Exercise‐induced plasma circAnks1b elevation is associated with superior recovery of neurological functions after IS. (A) Structure diagrams of human circAnks1b and Sanger sequencing, and agarose gel electrophoresis of PCR products with divergent primers (has‐circAnks1b) or convergent primers (Anks1b). (B) qRT‐PCR analysis of circAnks1b expression in rat plasma at four times (tMCAO+PE vs. tMCAO+Sed, *p* = 0.0008 at PE 4 W). (C) Correlation of mNSS score with rat circAnks1b expression in peri‐infarct cortex. (D) Correlation of infarct area and circAnks1b expression in peri‐infarct cortex. (E) The modified Barthel index to assess the neurological function of patients (*p* = 0.0240). (F) qRT‐PCR analysis of hsa‐circAnks1b in plasma of IS and IS+PE patients (*p* = 0.0068). (G) Correlation of the MBI score with hsa‐circAnks1b level determined by qRT‐PCR. (H) ROC curve for circAnks1b expression levels in IS and IS+PE patients. *p < 0.05, **p < 0.01, and ***p < 0.001. IS, ischemic; PE, physical exercise.

Finally, we examined if serum circAnks1b expression is associated with stroke prognosis as circRNAs are considered potentially advantageous biomarkers. For this purpose, we first examined the conservation of circAnks1b. CircAnks1b in human (hsa‐circ‐0096921) is highly homologous (87.27%) with that in rats (Figure S5A). Through Sanger sequencing, we confirmed the same BSJ in both rat and human circAnsk1b, and verified its cyclic structure by agarose gel electrophoresis (Figure 7A). Plasma circAnks1b was reduced after tMCAO surgery but significantly upregulated by PE (Figure 7B, tMCAO+PE vs. tMCAO+Sed group, *p* = 0.0008 at D28). There was also an inverse correlation between mNSS score and circAnks1b level in plasma (Figure 7C), and between infarct area and plasma circAnk1b (Figure 7D), consistent with the utility of plasma circAnsk1b as a prognostic biomarker. In human IS patients as well, exercise training improved neurological function compared to sedentary controls as evidenced by the MBI rehabilitation outcome scale at discharge (Figure 7E, *p* = 0.0240), and plasma circAnks1b was significantly higher in the PE group (Figure 7F, *p* = 0.0068). There was also a positive correlation between the MBI and plasma circAnks1b concentration in the stroke patients, (Figure 7G), and receiver operating characteristic (ROC) curve analysis indicated that high serum circAnks1b due to PE predicted better outcome with 88.90% sensitivity and 91.70% specificity (AUC = 0.9352, Figure 7H). Thus, upregulated plasma circAnks1b may serve as a biomarker for superior IS prognosis.

## 4 DISCUSSION

We demonstrate that circAnks1b upregulation is essential for the neuroprotection and improved neurological recovery conferred by PE in rats following tMCAO, a widely studied model of IS. Knockdown of circAnks1b diminished the beneficial effects of PE after IS, while overexpression in OGD/R‐exposed primary rat neurons reduced apoptosis. Furthermore, circAnks1b protected neurons from post‐IS apoptosis by acting as a miR‐130b‐5p sponge, leading to disinhibition of Pak2 expression, enhanced IRE1/XBP1 signaling, and activation of a cytoprotective ER stress mechanism. Upregulation of circAnks1b in plasma was also associated with better prognosis among IS patients. We conclude that the circAnks1b/miR‐130b‐5p/Pak2 axis is a promising therapeutic target for protecting neurons in the peri‐infarct region and accelerating neurological recovery post‐stroke.

PE is widely recommended for post‐stroke and neurodegenerative disease rehabilitation.^40^ One study comparing three groups of patients, exercise, social enrichment, and control, found that exercise cost‐effectively improved the cognitive function in elderly chronic stroke patients.^41^ Additionally, a Swedish study reported that increasing and sustaining light‐intensity physical activity during the subacute post‐stroke phase resulted in better functional outcomes at 6 months,^42^ consistent with both our rat model study and patient trial.

While it is well established that PE enhances neuronal survival in the peri‐infarct cortex,^4^, ^17^, ^19^ the precise protective mechanisms remain unclear. Non‐coding RNAs have a myriad of protective and pathogenic functions. Of these species, circRNAs are expressed at high abundance, are particularly stable, and highly conserved among mammals, leading to speculation that circRNAs may be valuable protective factors, biomarkers, and therapeutic targets for stroke.^36^, ^43^ While there is little evidence that PE beneficially alters the expression levels of specific circRNAs in the nervous system, one study found that aerobic exercise enhanced cognitive ability by reducing neuronal apoptosis via the circRIMS2/miR‐186/BDNF pathway in a VCI model,^10^ suggesting that PE‐induced circRNAs may promote rehabilitation from cerebrovascular diseases. Therefore, we investigated the possible types of PE‐induced circRNAs during stroke rehabilitation. Through high‐throughput sequencing and experimental verification, we identified circAnks1b as significantly elevated after PE in peri‐infarct cortex, and found that disruption of circAnks1b expression by shRNA transfection led to poor neurological recovery despite PE intervention.

CircRNAs serve various functions including inhibiting protein expression by sponging miRNAs, modulating protein activity, and in some cases encoding proteins.^36^ CircRNAs competitively bind to specific miRNAs through miRNA response elements (MREs).^43^ We found that circAnks1b was located mainly in the cytoplasm, consistent with a previous study,^37^ and that upregulation reduced miR‐130b‐5p through competitive binding as evidenced by RIP assays. Furthermore, we revealed that Pak2, which is predominantly expressed in neurons and also upregulated by PE, is a downstream target of miR‐130b‐5p. A recent investigation corroborated the presence of miR‐130b‐5p in HT‐22 neuronal cells following OGD/R,^44^ although the results further suggested that miR‐130b‐5p could be advantageous for neuronal survival. This discrepancy raises the possibility of distinct circRNA/miRNA/mRNA interactions among cell types and species.

Our primary result is that PE‐induced circAnks1b upregulation enhances the Pak2 expression via removal of miR‐130b‐5p suppression, resulting in protective ER stress through Pak2/IRE1a/ XBP1 signaling. We found that Pak2 is localized in close proximity to the ER membrane,^39^ and the ER is essential for intracellular protein synthesis, post‐translational modification, protein folding, and Ca^2+^ storage, all of which could be relevant to IS pathology. Cerebral ischemia/reperfusion can cause ER calcium dysregulation and the accumulation of misfolded proteins, termed ER stress, which in turn activates the UPR. Studies have demonstrated that inhibiting excessive UPR is conducive to recovery of nerve function, underscoring the significance of ER function restoration for stroke prognosis.^25^, ^45^ Although sustained or severe ER stress can trigger cell apoptosis via the CHOP and caspase‐3 pathways, moderate ER stress enhances cellular tolerance and aids in reestablishing cellular homeostasis.^46^ In our study, we found that PE elevated Pak2 expression in the peri‐infarct cortex post‐stroke, consistent with a study reporting that Pak2 enhances the IRE1/XBP1 branch of the UPR and thereby prevented heart failure.^39^ Our GO results also suggest that exercise training exerts regulatory effects on multiple ER‐related pathways, indicating that there may be other targets for the modulation of ER stress by PE. However, research on the modulation of ER stress by exercise is relatively scarce. One such study indicated that acute‐phase exercise post‐stroke increases SIRT1 expression, which regulates ER stress pathways involved in neuroprotection.^47^ In summary, our research suggests that during IS rehabilitation, moderate‐intensity exercise may convert prolonged post‐ischemic ER stress into protective ER stress by elevating Pak2.

Our findings reveal that circAnks1b upregulation by PE drives a neuroprotective response to ER stress through upregulation of Pak2. Furthermore, we demonstrated that circAnks1b is widely distributed in both the brain and plasma, emphasizing its broad potential physiological and pathogenic significance.^48^, ^49^ Rat circAnks1b showed a high degree of sequence homology with human circAnks1b (87.27%). Given these attributes, we believe circAnks1b has considerable potential as a biomarker, a drug delivery vehicle, and a therapeutic target. Consequently, we intend to explore the clinical potential of circAnks1b in IS rehabilitation. Moreover, we found consistent upregulation of circAnks1b in the plasma of ischemic patients. Some studies have demonstrated abnormal expressions of certain circRNAs, such as circOGDH, circFUNDC1, and circPDS5B, in AIS or cardiac disease.^16^, ^19^ These circRNAs, identified through screening of AIS patient plasma may also be potential therapeutic targets for these diseases. However, this is the first report on PE‐induced circRNA upregulation after IS, although a recent study reported that circUtrn is upregulated by exercise training and may serve as a new serum biomarker for cardiac disease prognosis.^11^ Given that PE is a systemic activity, we also speculate that circAnks1b may be a prognostic biomarker for IS. We found that circAnks1b expression in patient plasma was elevated after 4 weeks of PE, and that high levels distinguished above average from below average recovery with 91.7% specificity and 88.90% sensitivity, further underscoring the potential utility of circAnks1b as a prognostic biomarker for assessing the benefits of PE following stroke.

Our study also has several limitations. First, it is noteworthy that sexual dimorphism has been increasingly recognized to modify the neurovascular functions in healthy adults.^50^, ^51^ It influences lipid metabolism and relevant inflammatory signaling in pathological brain.^52^ Besides, there are sexual differences in response to disease‐modifying therapies for neurovascular disorders.^53^ Furthermore, stroke is a sexually dimorphic disease, with higher incidence in males, but higher mortality and severe symptoms in females upon occurrence.^54^ More literature suggested that the regulation of PE on energy metabolism and vascular adaptations are different between men and women,^55^, ^56^ but there are no sexual differences in the effects of exercise on memory, executive functioning, language or global cognition in individuals with stroke.^57^ We carried out tMCAO with or without PE entirely on male rats to examine the effects of exercise on IS. This is the limitation in our study, and we will explore sexual differences of exercise in the regulation of ER stress during IS in our future study. Second, more patients are needed to verify the significance of plasma circAnks1b for predicting exercise effects. Finally, we focused solely on circAnks1b, although there may be other protective circRNAs in plasma. CircRNAs are still not widely studied or targeted in clinical medicine, so the current study offers a valuable rationale for further studies on the pathological significance of circRNAs.

In conclusion, our findings highlight the crucial role of circAnks1b elevation in the neuroprotective effects of PE in tMCAO rats. We show that circAnks1b serves as a ceRNA sponge for miR‐130b‐5p, resulting in upregulation of Pak2, which in turn facilitates protective ER stress and alleviates neuronal apoptosis. This mechanism may be crucial for the rehabilitation potential of post‐stroke PE, and plasma circAnks1b elevation may be a possible treatment strategy for alleviating neurological dysfunction after IS.

## AUTHOR CONTRIBUTIONS

Xiaofeng Yang, Yating Mu, and Yifeng Feng performed the experiments and collected data, and they contributed equally to this work. Mingyue Li, and Xiaoya Zhang collected the samples from stroke patients. Xiaofeng Yang drafted the manuscript, Haojie Hu edited the manuscript. Zejie Zuo, and Rui Wu provided the necessary materials. Jinghui Xu and Fang Zheng contributed to the experimental work. Xiaofei He revised the manuscript, Xiquan Hu and Liying Zhang designed and organized the research project. All authors read the manuscript and approved the submission.

## CONFLICT OF INTEREST STATEMENT

All authors declared that they have no conflict of interests.

## Supporting information


**Data S1:** Supporting information. Please remove the highlighting from the supporting information.

## Data Availability

The data that support the findings of this study are available from the corresponding author upon reasonable request.
