# Supplementary material for: Physical exercise‐induced circAnks1b upregulation promotes protective endoplasmic reticulum stress and suppresses apoptosis via miR‐130b‐5p/Pak2 signaling in an ischemic stroke model
Source: CNS Neurosci Ther. 2024 Sep 27;30(9):e70055. doi: 10.1111/cns.70055 (PMC11427801; doi:10.1111/cns.70055)
Supplement: Supplementary file 1 — Data S1: Supporting information. Please remove the highlighting from the supporting information. [file CNS-30-e70055-s001.zip › 3. Supplemental Information.docx]

**Supplemental Information**

**Physical exercise-induced circAnks1b upregulation promotes protective endoplasmic reticulum stress and suppresses apoptosis via miR-130b-5p/Pak2 signaling in an ischemic stroke model**

Xiaofeng Yang^a#^, Yating Mua^#^, Yifeng Feng^a#^, Mingyue Li^a^, Haojie Hu^b^, Xiaoya Zhang^a^, Zejie Zuo^a^, Rui Wu^a^, Jinghui Xu^a^, Fang Zheng^a^, Xiaofei He^a^*, Xiquan Hu^a^*, Liying Zhang^a^*


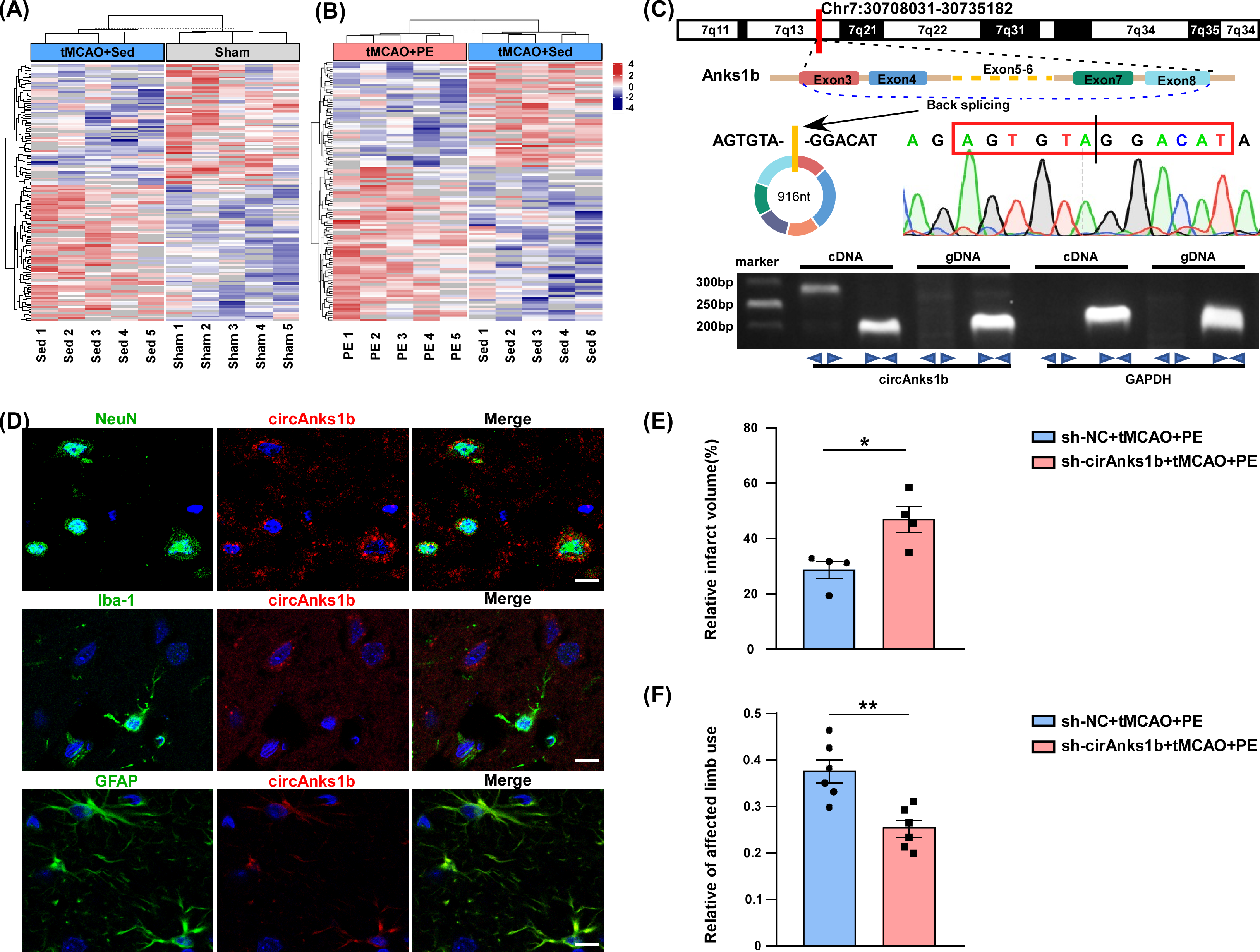


**Figure S1, related to Figure 1.** (A) Heatmap plot of circRNAs between Sham and tMCAO+Sed group. (B) Heatmap plot of circRNAs between tMCAO+PE and tMCAO+Sed group. (C) Structure diagrams of rat circAnks1b and Sanger sequencing (up); Agarose gel electrophoresis was utilized to analyze PCR products amplified with divergent primers (rno-circAnks1b) or convergent primers (Anks1b) (down). (D) FISH assay showed the expression of circAnks1b and neurons (NeuN), microglia (Iba1) and astrocytes (GFAP) in peri-infarct cortex. (E) TTC staining of brain tissues after sh-NC or sh-circAnks1b treatment, and quantification of brain infarcts (*p* = 0.0203). (F) The long-term effect on motor functions of stroke-damaged rats measured by cylinder test (*p* = 0.0026). Scale bar is representative of 10 μm (D).


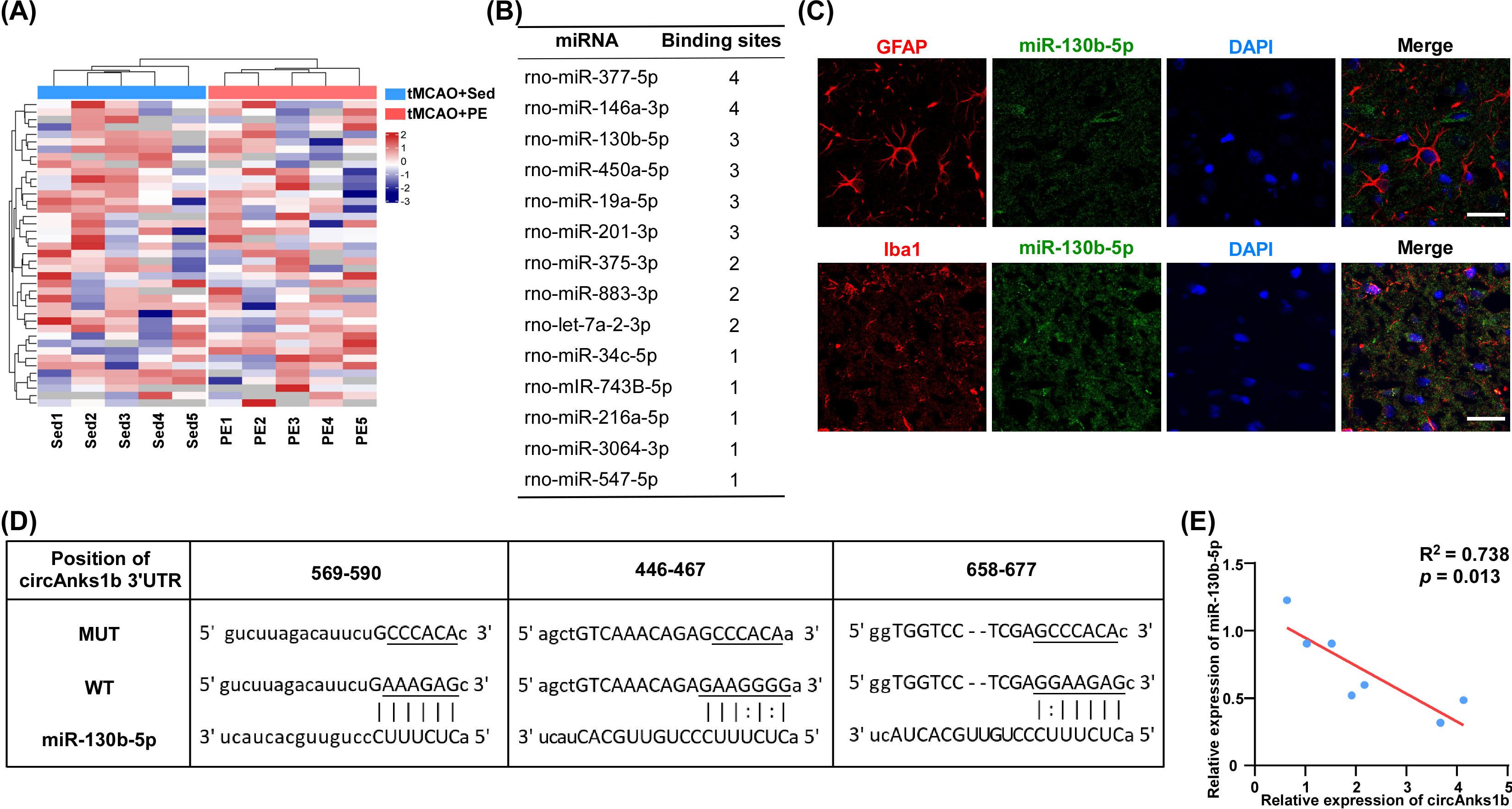


**Figure S2, related to Figure 2.** (A) Heatmap plot of miRNAs between tMCAO+PE and tMCAO+Sed group. (B) The binding sites of circAnks1b and the 14 miRNAs screened from venn diagram. (C) FISH assay revealed the expression of miR-130b-5p and microglia (Iba1) and astrocytes (GFAP) in peri-infarct cortex. (D) The three sites of circAnks1b binding with miR-130b-5p wild-type (WT) and mutant (MUT). (E) Correlation analyses of circAnks1b and miR-130B-5p collected from OGD/R primary neurons. Scale bar is representative of 20 μm (C).


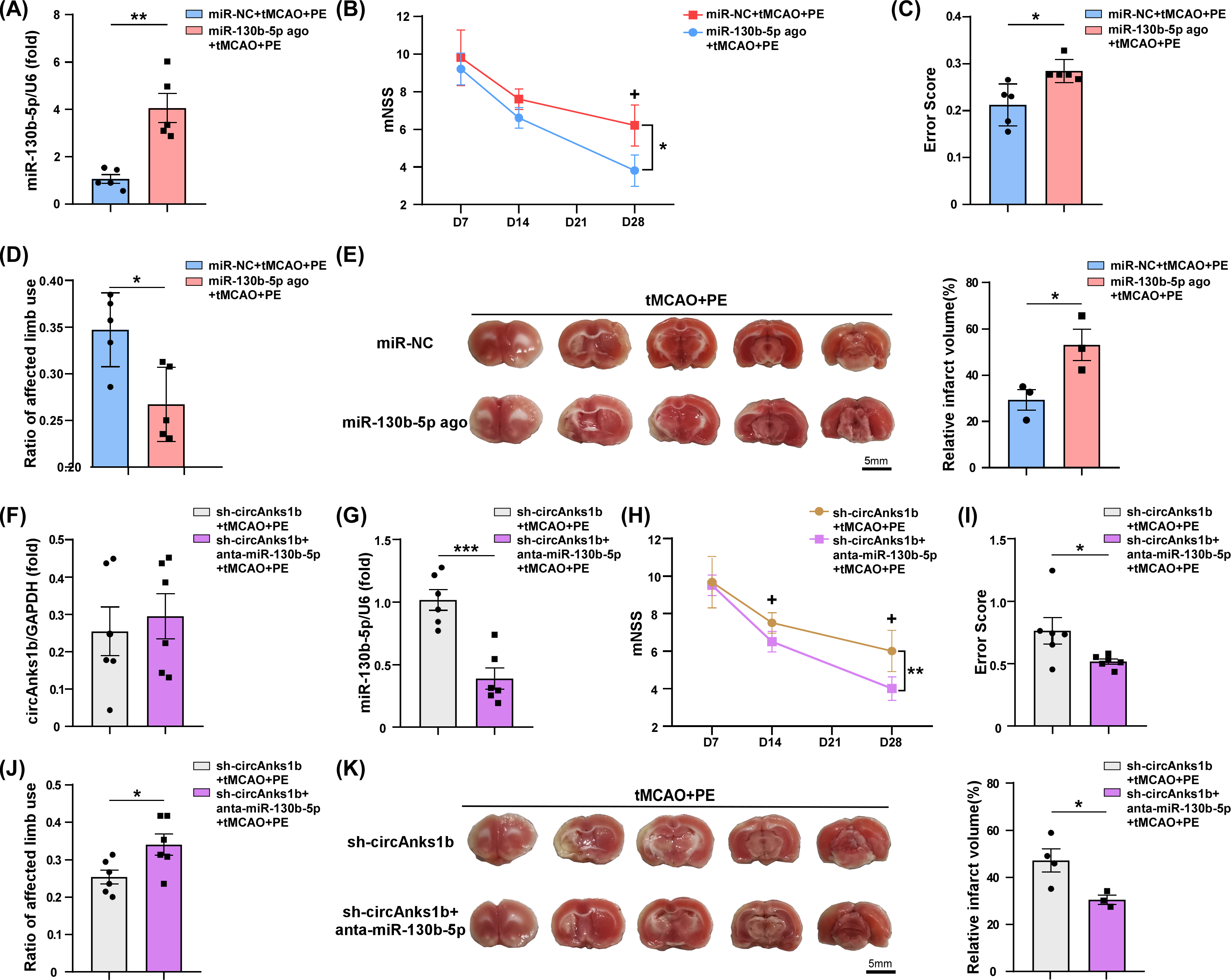


**Figure S3. CircAnks1b serves as a sponge for miR-130b-5p, reducing its levels post-exercise in the peri-infarct cortex.** (A). qRT‐PCR analysis of miR-130b-5p in peri-infarct cortex (*p* = 0.0016). (B-D) Neurological function was measured by mNSS (B, *p* = 0.0156 at D28), the ladder rung walking task (C, *p* = 0.0133), and cylinder test (D, *p* = 0.0129). (E) TTC staining of brain tissues after miR-NC or miR-130b-5p agonist treatment, and quantification of brain infarcts (*p* = 0.0432). (F-G) qRT‐PCR analysis of circAnks1b, miR-130b-5p in peri-infarct cortex (G, *p* = 0.0004). (H-J) Neurological function was measured by mNSS (H, *p* = 0.0141 at D28), the ladder rung walking task (I, *p* = 0.0471), and cylinder test (J, *p* = 0.0293). (K) TTC staining of brain tissues after sh-circAnks1b or sh-circAnks1b plus anta-miR-130b-5p treatment, and quantification of brain infarcts (*p* = 0.0393). **p* < 0.05, ***p* < 0.01, ****p* < 0.001; ^+^*p* < 0.05, tMCAO+PE vs tMCAO+Sed group at different time point.


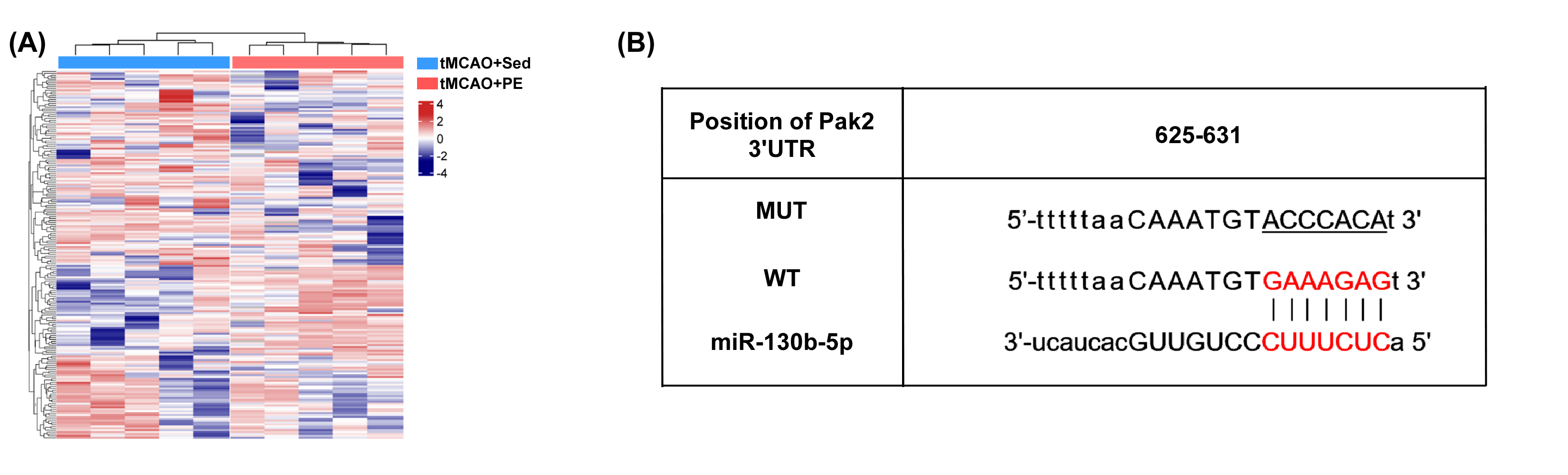


**Figure S4, related to Figure 3.** (A) Heatmap plot of mRNAs between tMCAO+PE and tMCAO+Sed group. (B) The binding site of Pak2 with miR-130b-5p wild-type (WT) and mutant (MUT).


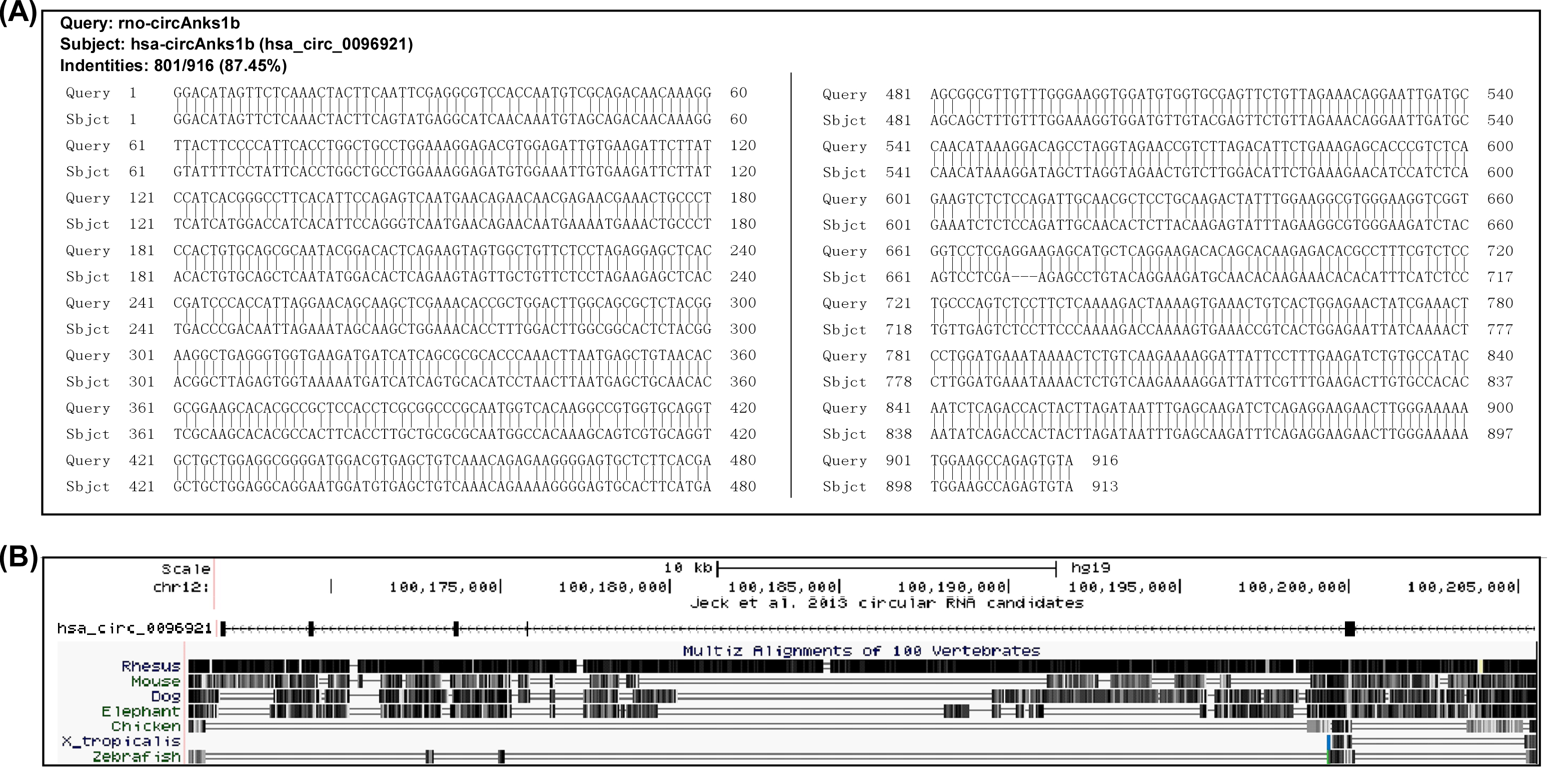


**Figure S5, related to Figure 7.** (A) BLAST analysis of rno-circAnks1b and hsa-circAnks1b sequences from NCBI blast websites. (B) hsa-circAnks1b conservation analysis compared with rat genomes in UCSC schematic drawing.

**Supplementary Table 1. Sequences of the primers used in the study**

| **Name** | **F/R** | **Sequence (5’-3’)** |
| --- | --- | --- |
| rno-circAnks1b | F | TTCCTTTGAAGATCTGTGCCA |
|  | R | TCACAATCTCCACGTCTCCT |
| hsa-circAnks1b | F | TGCCACACAATATCAGACCAC |
|  | R | CCAGGCAGCCAGGTGAATA |
| hsa-GAPDH | F | GTCTCCTCTGACTTCAACAGCG |
|  | R | ACCACCCTGTTGCTGTAGCCAA |
| rno-miR-130b-5p | RT | GTCGTATCCAGTGCAGGGTCCGAGGTATTCGCACTGGATACGACAGTAGT |
|  | F | CGCGACTCTTTCCCTGTTGC |
|  | R | AGTGCAGGGTCCGAGGTATT（the same as other miRNAs） |
| rno-miR-146a-3p | RT | GTCGTATCCAGTGCAGGGTCCGAGGTATTCGCACTGGATACGACAAAGAA |
|  | F | CGCAGACCTGTGAAGTTCAG |
| rno-miR-377a-5p | RT | GTCGTATCCAGTGCAGGGTCCGAGGTATTCGCACTGGATACGACGAATTC |
|  | F | CCAGAGGTTGCCCTTGGT |
| rno-U6 | F | CCTGCTTCGGCAGCACA |
|  | RT/R | AACGCTTCACGAATTTGCGT |
| rno-Pak2 | F | TTTCCTCTCTGGCCGTTTCC |
|  | R | ATGCTCAAAATCAGACGGCG |
| rno-GAPDH | F | TGTTCTAGAGACAGCCGCATC |
|  | R | TCCCGTTGATGACCAGCTTC |
| rno-GRP78 (Hspa5) | F | CATAGCCAACGATCAGGGCA |
|  | R | AAGGGTCATTCCAAGTGCGT |
| rno-IRE1a | F | GCAGTTCCAGTACATTGCCATTG |
|  | R | CAGGTCTCTGTGAACAATGTTGA |
| rno-XBP1 | F | CTGAGTCCGCAGCAGGTG |
|  | R | GCTCTCTGTCTCAGAGGGGA |
| rno-Anks1b | F | TTTGGGAAGGTGGATGTGGT |
|  | R | TCCCACGCCTTCCAAATAGT |
| rno-dGAPDH | F | CTTCCACCTTTGATGCTGGG |
|  | R | CACACCGACCTTCACCATTTA |
