# Supplementary material for: Physical exercise‐induced circAnks1b upregulation promotes protective endoplasmic reticulum stress and suppresses apoptosis via miR‐130b‐5p/Pak2 signaling in an ischemic stroke model
Source: CNS Neurosci Ther. 2024 Sep 27;30(9):e70055. doi: 10.1111/cns.70055 (PMC11427801; doi:10.1111/cns.70055)

Full unedited gel/blot for Figure 3E

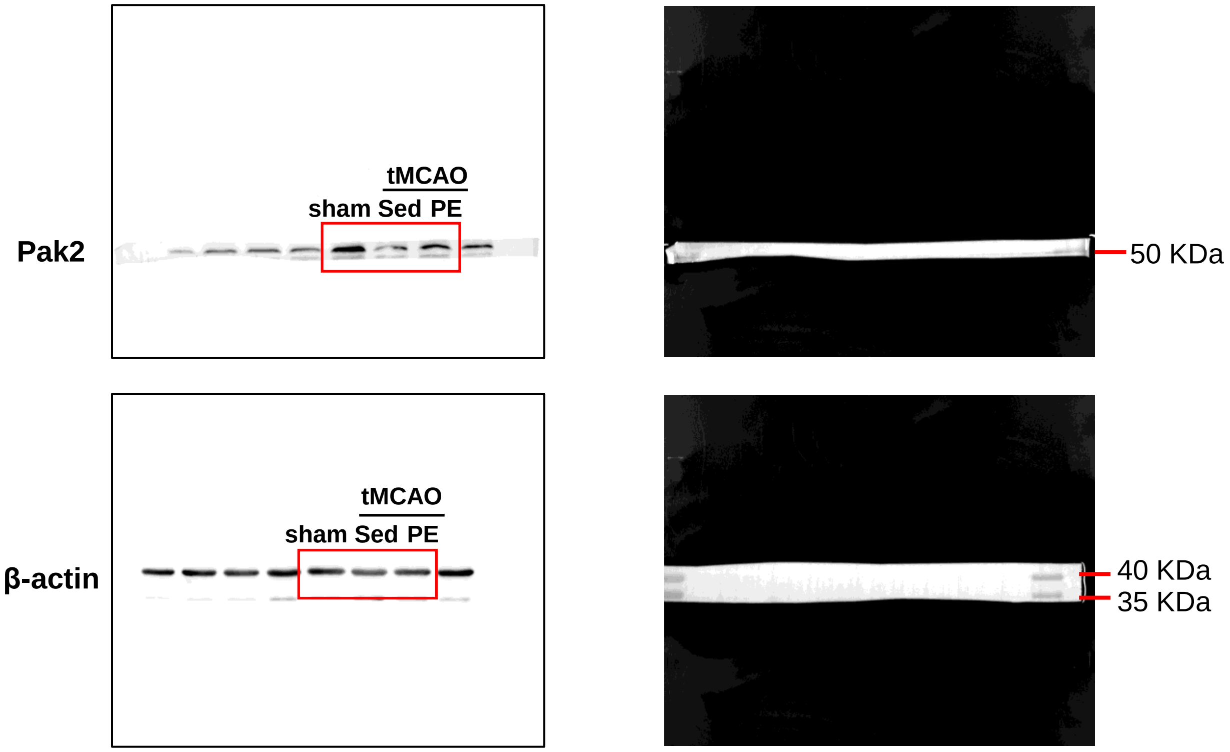

Full unedited gel/blot for Figure 4A

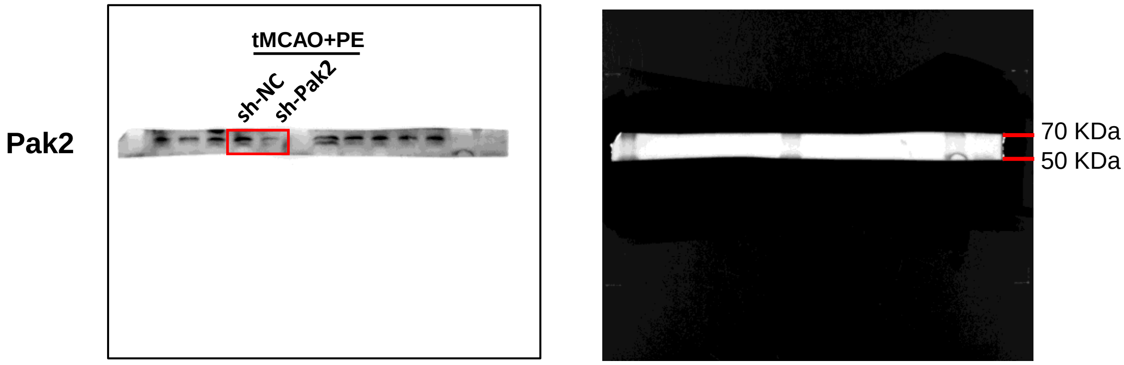

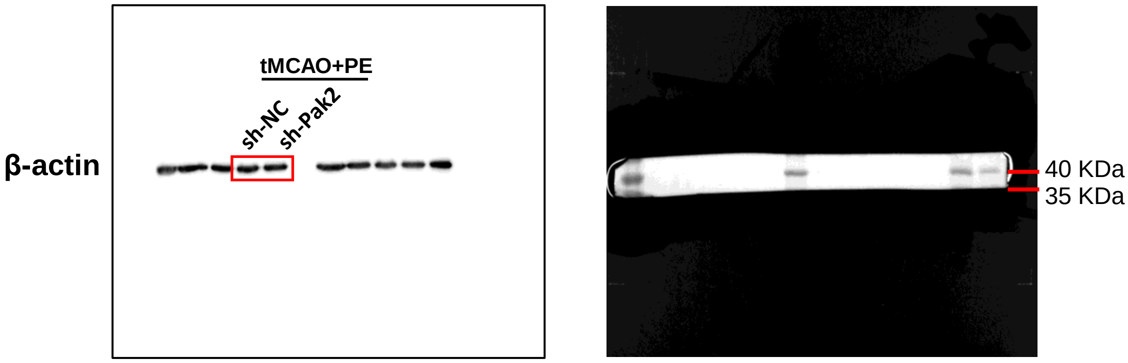

Full unedited gel/blot for Figure 4G

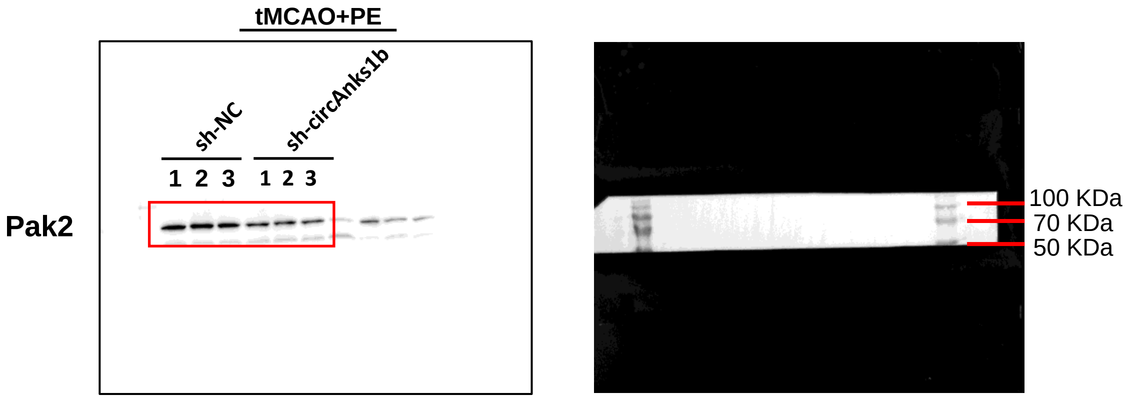

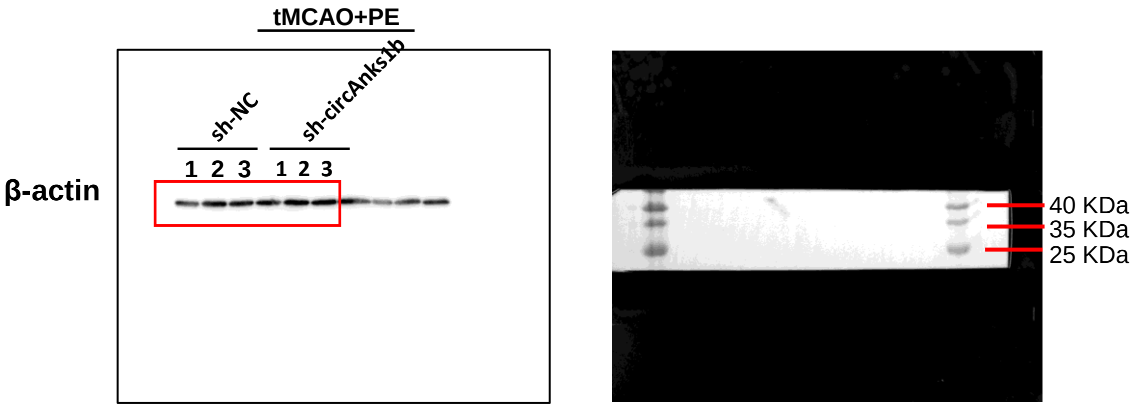

Full unedited gel/blot for Figure 4K

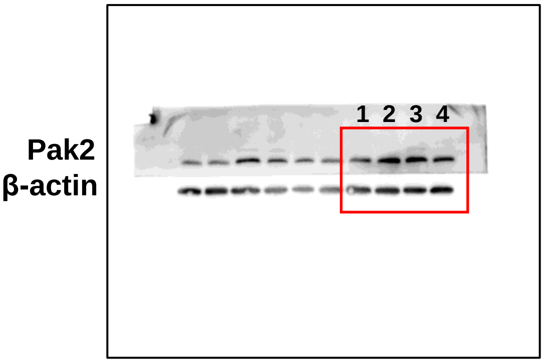

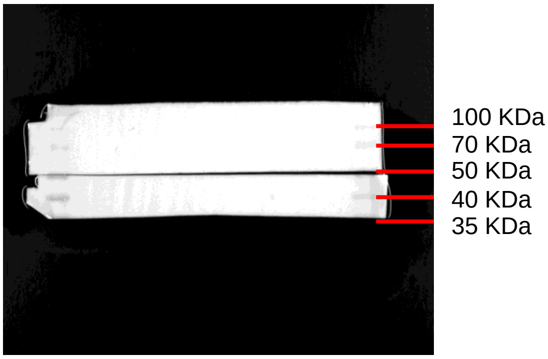

1. Vector
2. OE-circAnks1b
3. miR-130b-5p inhibitor
4. OE-circAnks1b+130b mimic

Full unedited gel/blot for Figure 5C

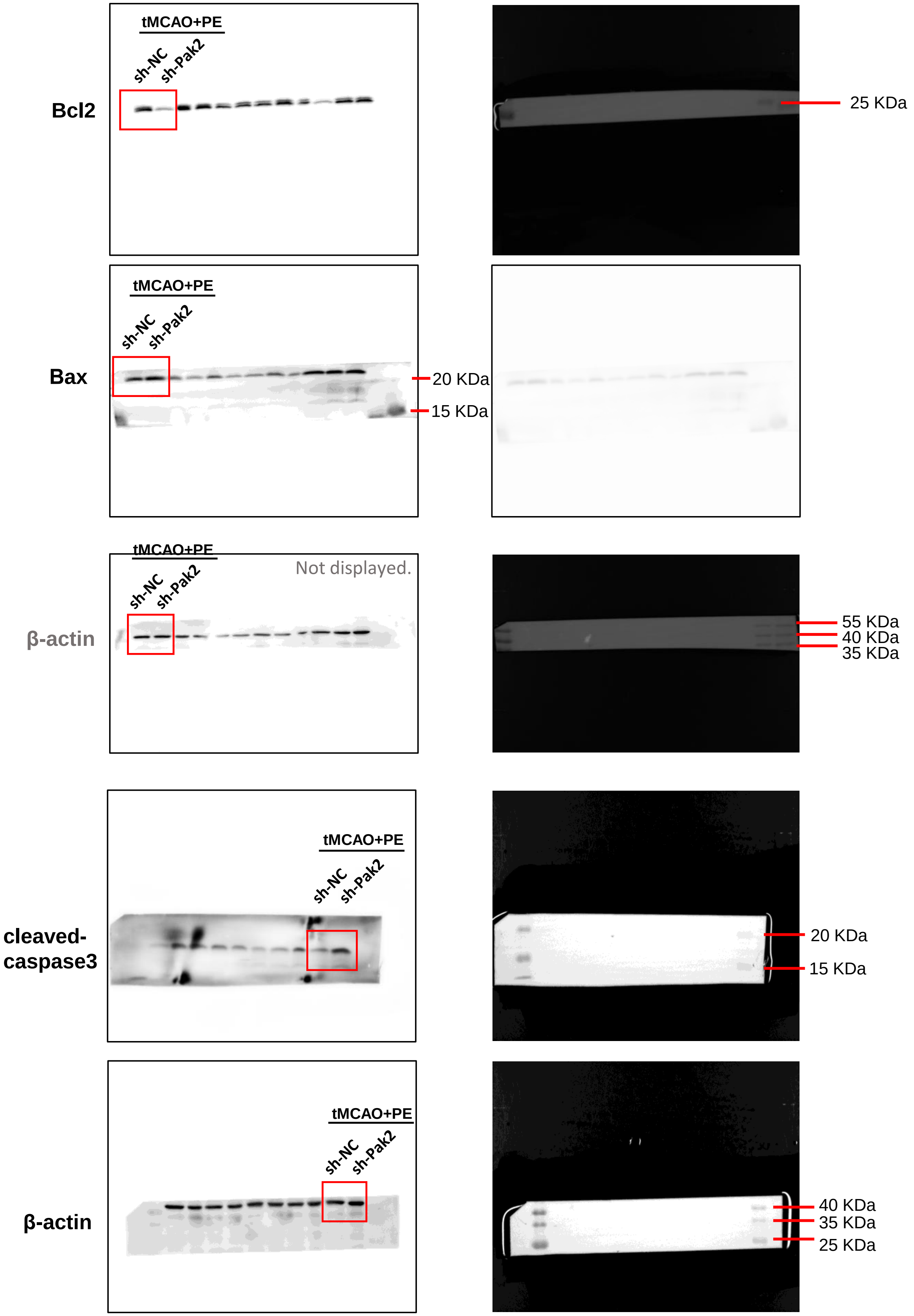

Full unedited gel/blot for Figure 5F

Bcl2

Bax

cleaved-caspase3

β-actin

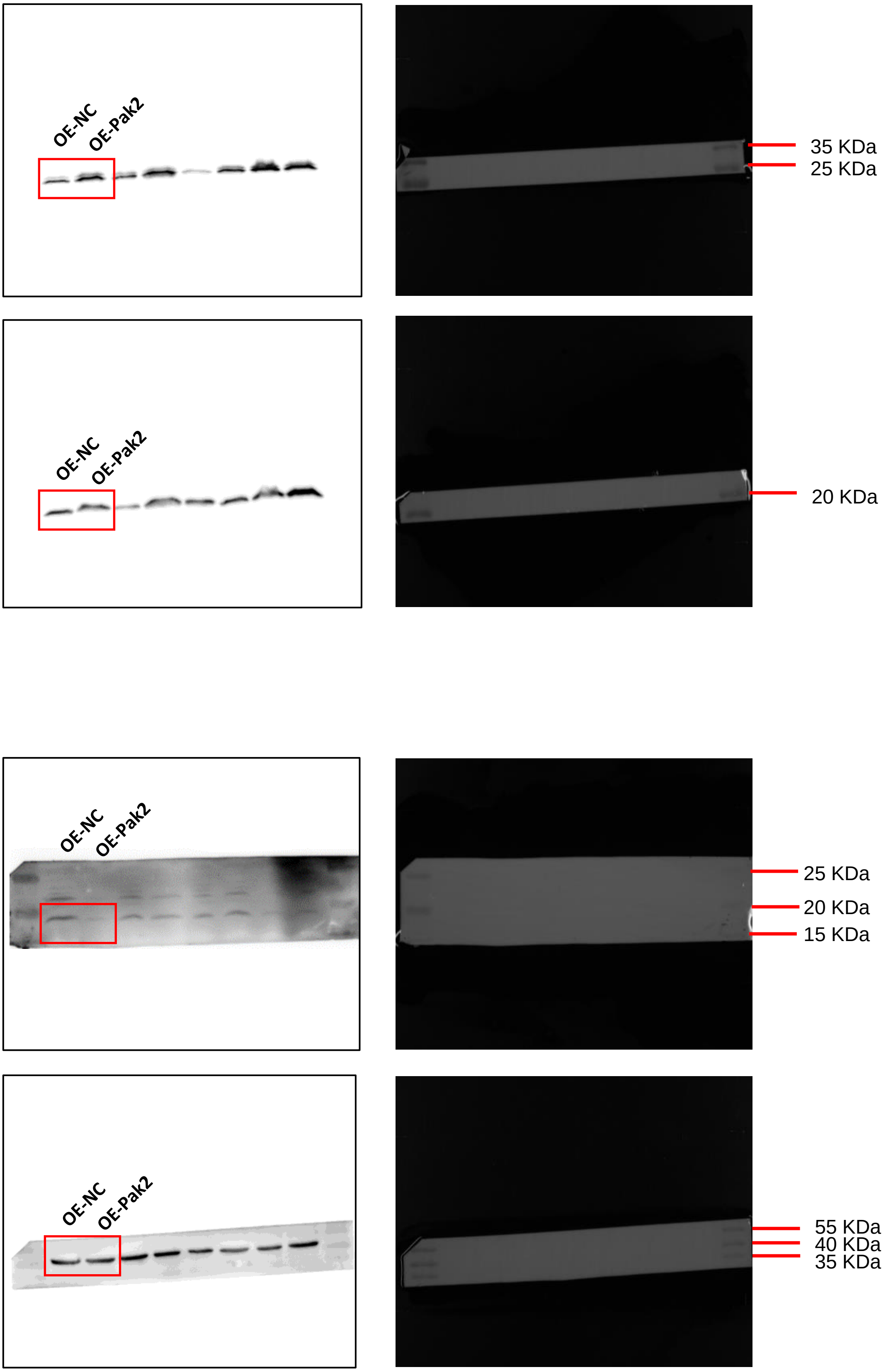

Full unedited gel/blot for Figure 6A

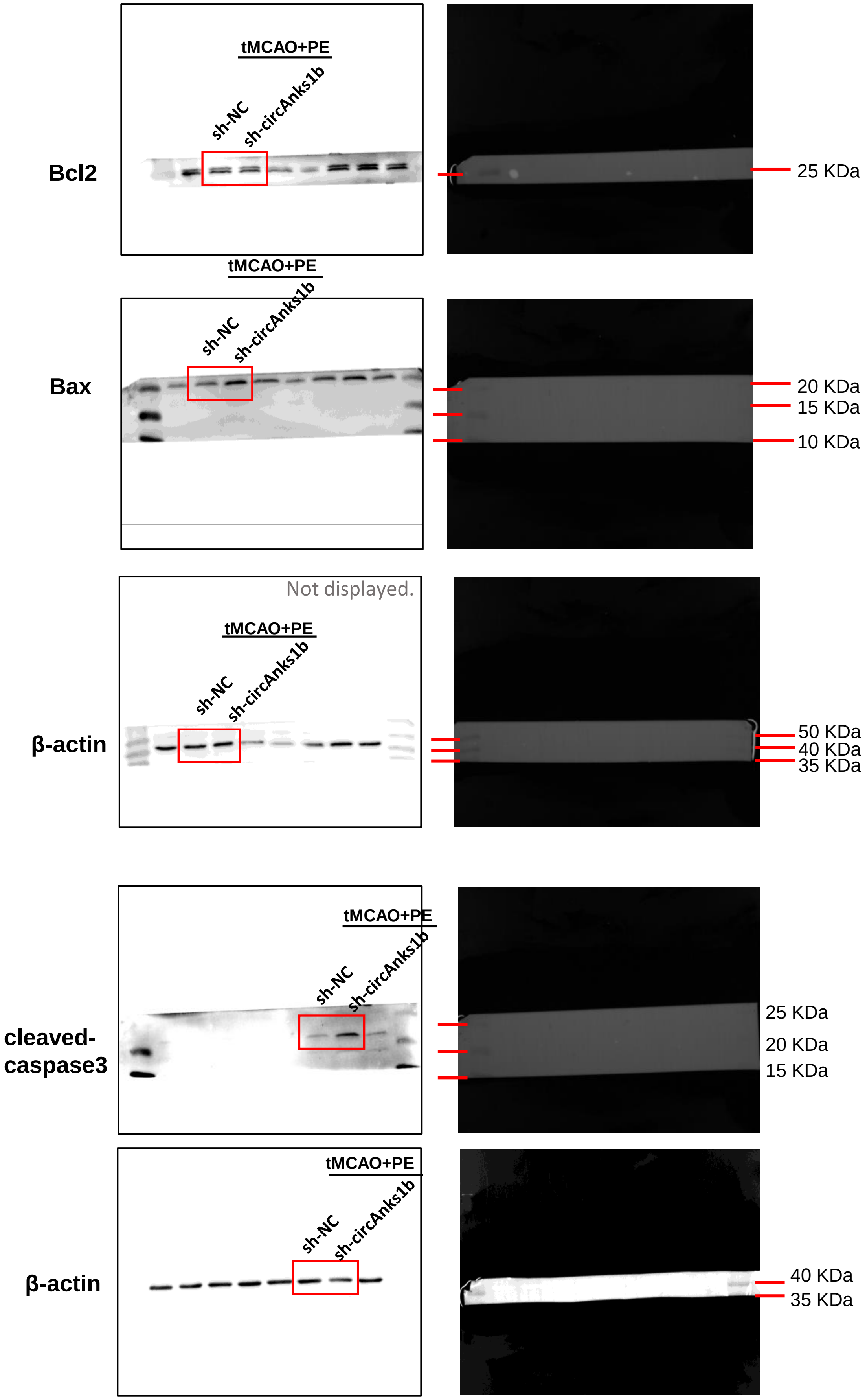

Full unedited gel/blot for Figure 6C

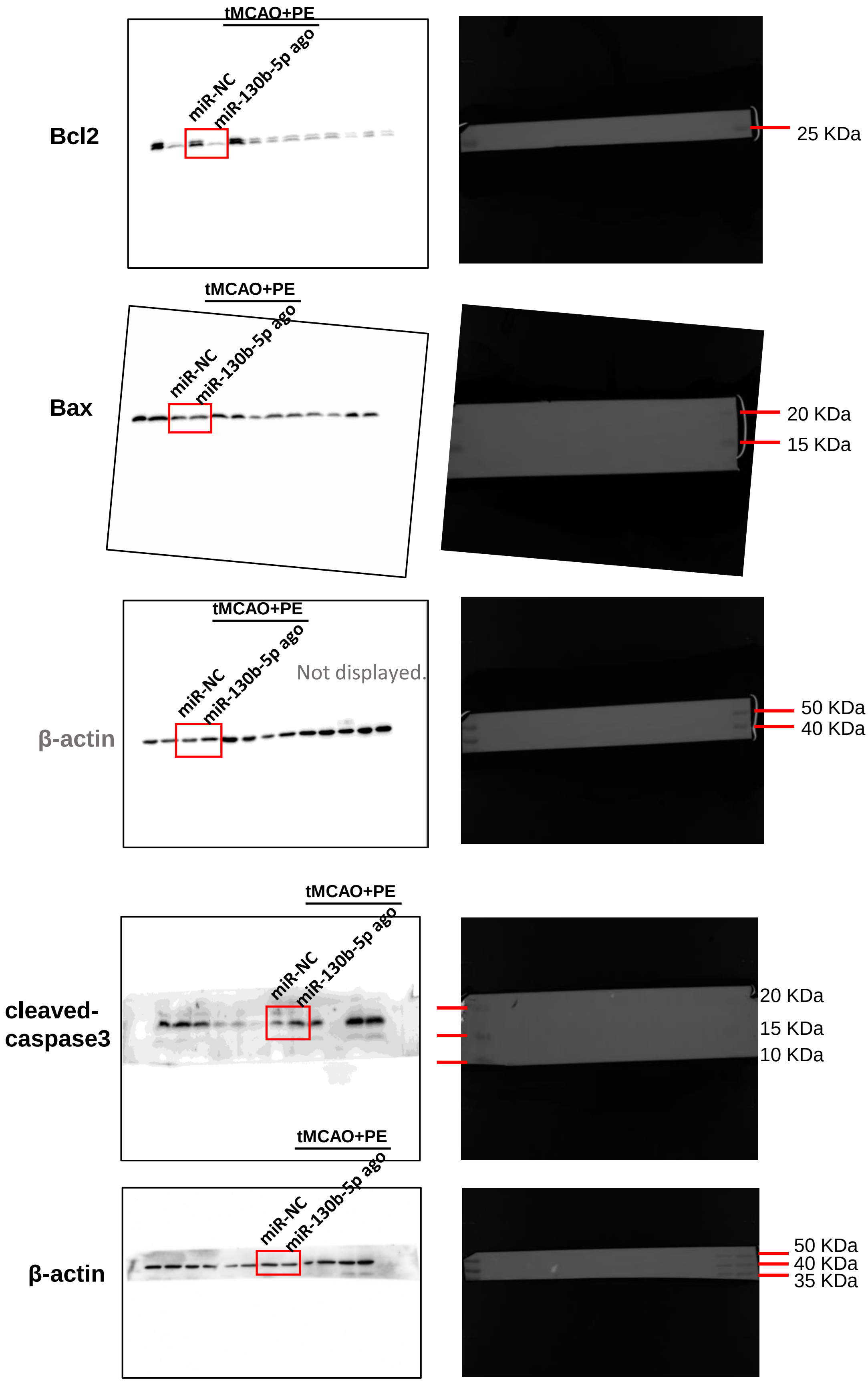

Full unedited gel/blot for Figure 6E

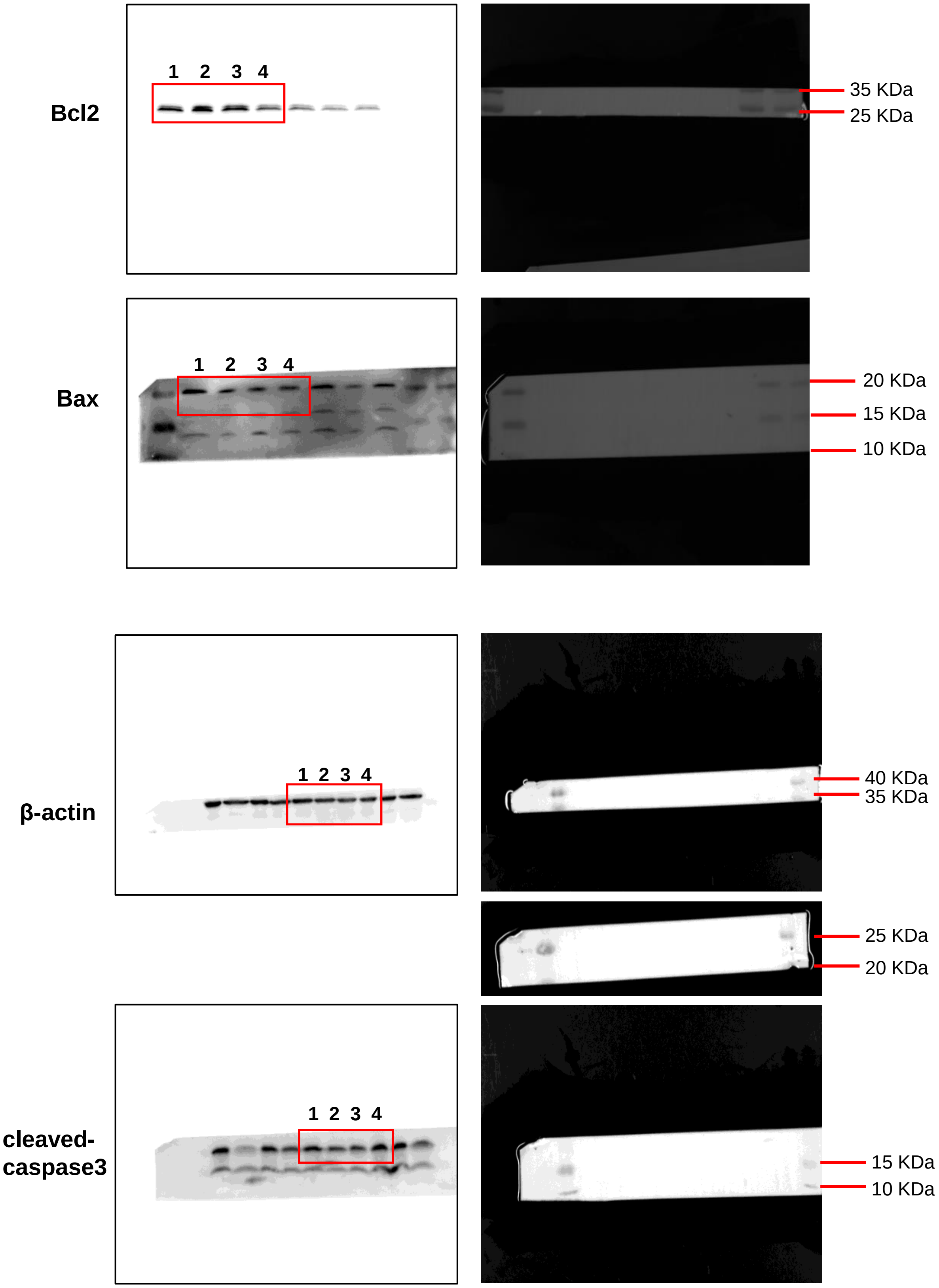

1. **Vector**
2. **OE-circAnks1b**
3. **miR-130b-5p inhibitor**
4. **OE-circAnks1b+130b mimic**

Full unedited gel/blot for Figure 7A

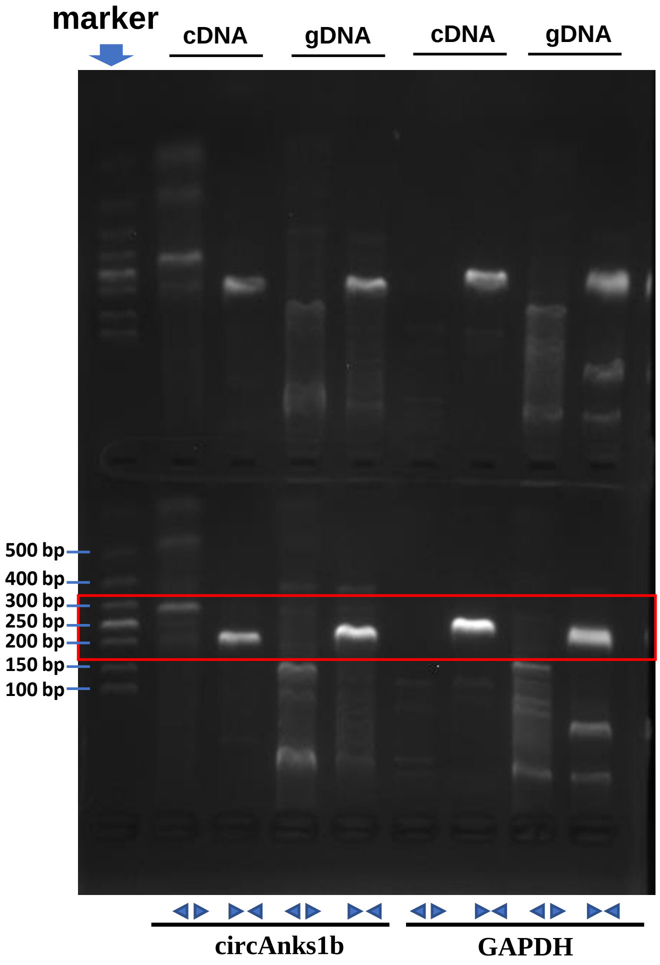

Full unedited gel/blot for Figure S1C

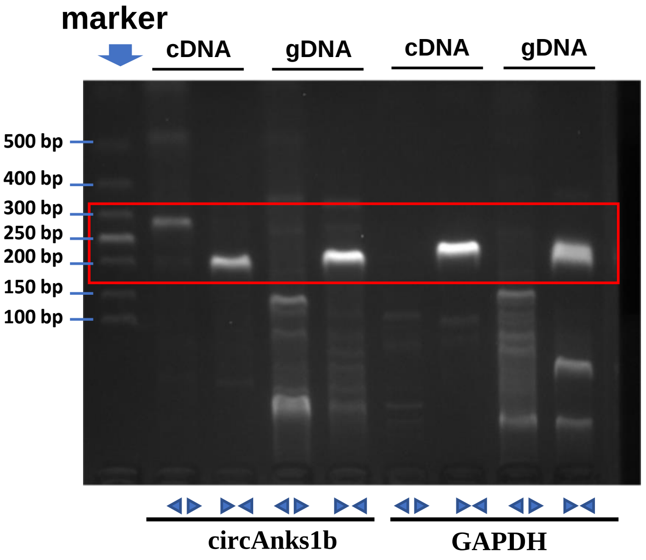

Supplement: Supplementary file 1 — Data S1: Supporting information. Please remove the highlighting from the supporting information. [file CNS-30-e70055-s001.zip › Images of full unedited gel and blot.pdf]
